# The entire *CYP51B* locus in azole-resistant isolates of the dermatophyte *Trichophyton indotineae* revealed by optical genome mapping

**DOI:** 10.1128/aac.01817-25

**Published:** 2026-03-31

**Authors:** Tsuyoshi Yamada, Mari Maeda, Minami Nakagawa, Takashi Yaguchi, Masaki Ishii, Karine Salamin, Christine Pich-Bavastro, Michel Monod

**Affiliations:** 1 Teikyo University Institute of Medical Mycology, Tokyo, Japan; 2 Asia International Institute of Infectious Disease Control, Teikyo University, Tokyo, Japan; 3 Research Center, Research Division, Nihon Nohyaku Co., Ltd., Osaka, Japan; 4 Medical Mycology Research Center, Chiba University 118076, Chiba, Japan; 5 Faculty of Pharmacy, Research Institute of Pharmaceutical Sciences, Musashino University, Tokyo, Japan; 6 Department of Dermatology, Centre Hospitalier Universitaire Vaudois 30635, Lausanne, Switzerland; 7 Faculty of Biology and Medicine, University of Lausanne 30588, Lausanne, Switzerland; University Children's Hospital Münster, Münster, Germany

**Keywords:** dermatophytes, *Trichophyton indotineae*, antifungal resistance, *TinCYP51B*, gene duplication, optical genome mapping, polycistronic/monocistronic gene expression

## Abstract

The resistance of *Trichophyton indotineae* to azoles is mainly due to the overexpression of *TinCYP51B,* resulting from additional copies of this gene in two types of strains (type I and type II). Due to its large size and the significant number of duplicated blocks, whole-genome sequencing has been unable to cover the entire *TinCYP51B* locus. Through optical genome mapping (OGM), we have successfully determined the copy number of the *TinCYP51B* gene in the genomes of resistant strains. The *TinCYP51B* copy number was lower in the type I strains than in the type II strains, while the *TinCYP51B* expression level was higher in the type I strains. To explain this paradox, we have revealed that polycistronic transcription of multiple *TinCYP51B* open reading frames (ORFs) alongside monocistronic transcription occurs in type I azole-resistant strains. In contrast, type II strains generated only the transcripts encoding one CYP51B polypeptide. OGM has also revealed that a 970 kb region on chromosome 3 is inverted in type I strains and the azole-susceptible strain TIMM20115, as compared to type II strains and the azole-susceptible strain TIMM20114. This has led to the hypothesis that under azole stress, type I resistant strains originate from susceptible strains such as TIMM20115, which possesses a single *TinCYP51B* gene. Conversely, it is believed that type II azole-resistant strains evolve from susceptible strains such as TIMM20114, which also has only one *TinCYP51B* gene. In conclusion, strains of *Trichophyton indotineae* can be divided into two groups in which a distinct type of resistance has developed.

## INTRODUCTION

*Trichophyton indotineae* is an emerging dermatophyte that causes severe tinea corporis and tinea cruris (dermatophytosis), many of which are resistant to terbinafine and/or azole compounds (1–3). In all cases of dermatophytoses, terbinafine resistance is due to point mutations in the gene encoding squalene epoxidase (*SQLE*) in the ergosterol synthesis pathway, leading to amino acid substitutions at one of the five amino acid positions (Leu393, Phe397, Gln408, Phe415, and His440) in the enzyme (1, 4). In contrast, the reduced susceptibility of *T. indotineae* to azoles is mainly due to the overexpression of the *CYP51B* gene (*TinCYP51B*) encoding a sterol 14α-demethylase, a target of azole compounds such as itraconazole (ITC) and voriconazole (VRC) in the same ergosterol synthesis pathway (5). We recently found that the overexpression of *TinCYP51B* resulted from additional copies of this gene in tandem in two types of strains (types I and II) (6). Type I strains harbor *TinCYP51B* duplications with 2,404 bp end-to-end blocks containing only this gene. In contrast, the genome of the type II strains contains large segmental duplications of 7,374 bp. These duplications contain *TinCYP51B*, as well as two adjacent genes: *TinCHBK*, which encodes upstream a homolog of the *Aspergillus nidulans* chkB kinase (AN4279) (7, 8), and *TinFYV4,* which encodes downstream a homolog of the *Saccharomyces cerevisiae* FYV4 (YHR059W) and is part of the small subunit of the mitochondrial ribosome (9). The overexpression of the gene encoding the drug target is an effective mechanism for acquiring resistance. The effect of the drug is countered by a disruption of the drug-target balance in favor of the target.

The existence of multiple copies of the *TinCYP51B* gene in tandem within the genomes of types I and II azole-resistant strains was revealed through whole-genome sequencing (WGS) using PacBio long-read technology. However, technological limitations prevented this technique from revealing the exact number of duplications in the genomes of *T. indotineae* resistant strains. Due to the large size of the duplicated blocks and their multiplicity (the fact that there were at least five copies of the duplicated block), the single PacBio reads could not cover the entire *TinCYP51B* locus. The copy number of the *TinCYP51B* gene was then estimated by qPCR analysis with genomic DNA (gDNA) used as a template. Combining the results of the PacBio sequencing and qPCR, it was estimated that there were between 5 and 14 copies of *TinCYP51B* (5, 6).

In this report, we tried to determine the exact *TinCYP51B* copy number by OGM. Since the first description of chromosome mapping in baker’s yeast in the early 1990s (10), this technique (initially termed optical mapping) has undergone repeated updates from various angles, evolving into an important auxiliary tool for genome research across a wide range of organisms, from microorganisms to humans (11–15). It is now used for assembly and validation of WGS using high-throughput sequencing methods, and comparative genomic profiling based on the detection of structural variations (SVs) in genomes. OGM is based on imaging ultra-long single DNA molecules (over 150 kb) that have been fluorescently labeled at specific sequence motifs throughout the genome. Comparison of the labeling pattern to a reference genome enables the detection of different classes of SVs, including triploidy, aneuploidies (insertions, inversions, and translocations) at high resolution that are undetectable or hard-to-detect via short- and long-read sequencing approaches. By using OGM, we were able to determine the copy number of the *TinCYP51B* gene in the genomes of types I and II azole-resistant strains. In addition, OGM revealed a genomic inversion of approximately 970 kb located downstream of the *TinCYP51B* locus in type I azole-resistant *T. indotineae* strains compared to type II strains. Paradoxically, the *TinCYP51B* copy number was lower in the type I strains than in the type II strains, while the *TinCYP51B* overexpression was higher in the type I strains than in the type II strains. We showed that duplications of the *TinCYP51B* gene were more efficient in the type I resistant strains than large segmental duplications in the type II strains for overexpressing *TinCYP51B*.

## RESULTS

To further investigate the mechanisms involved in azole resistance in *T. indotineae*, we focused on two azole-sensitive strains, TIMM20114 and TIMM20115, that have a MIC80 ≤ 0.06 µg/mL for ITC and a MIC80 ≤ 0.03 µg/mL for VRC, three type I azole-resistant strains, TIMM20118, TIMM20119, and IFM66168, and two type II azole-resistant strains, TIMM20121 and TIMM20122, that have ITC MIC_80_ ≥ 0.5 µg/mL and VRC MIC_80_ ≥ 0.5 µg/mL (Table 1).

**TABLE 1:** Phenotypic and genotypic characteristics of *T. indotineae* strains used in this study

*T. indotineae* strains^*a*^	ITC MIC_80_ (µg/mL)	VRC MIC_80_ (µg/mL)	Strain type
TIMM20114 (**UKJ1676/17**; IFM67092)	0.06	0.015	1 *TinCYP51B* gene
TIMM20115 (**UKJ1700/17**; IFM 67093)	0.06	0.03	1 *TinCYP51B* gene
IFM66168 (**NUB19006^T^**)	0.5	0.5	I
TIMM20118 (**UKJ1687/18**; IFM67096)	0.5	1.0	I
TIMM20119 (**200123/18**; IFM67097)	1.0	1.0	I
TIMM20121 (**250084/18**)	0.5	1.0	II
TIMM20122 (**250108/18**)	0.5	0.5	II

^*a*^ IFM66168 is the type strain of *Trichophyton indotineae* (16). This strain originates from Japan. All other strains were from a previously published resistance study in India, with the numbering in bold (1). All the strains were then preserved in the culture collection of Teikyo University Institute of Medical Mycology (TIMM) and/or Medical Mycology Research Center, Chiba University (IFM), through the National Bio-Resource Project, Japan (http://www.nbrp.jp/). ITC, itraconazole; VRC, voriconazole.

### Optical genome mapping with genome imaging revealed the exact copy number of *TinCYP51B* genes in the azole-resistant *T. indotineae* strains

**Fig 1:**
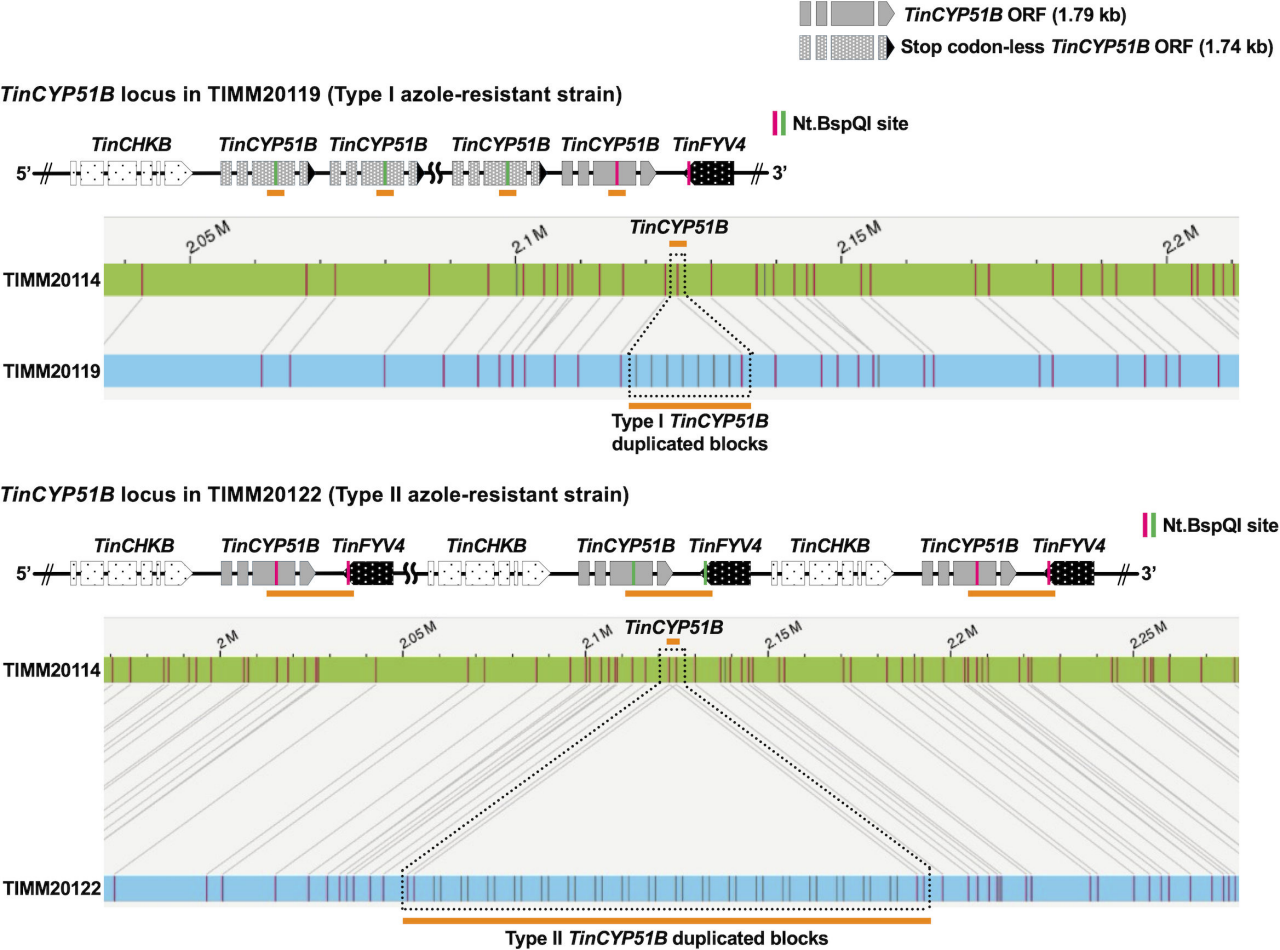
Two types of *TinCYP51B* gene duplications in tandem visualized by OGM. The green bars show chromosome 3 (the contig 2, ctg.000002F) of TIMM20114 used as a reference sequence, and the blue bars show the maps assembled from the OGM data of TIMM20119 and TIMM20122. The vertical pink and green lines on the bars indicate the location and pattern of the GCTCTTG motif recognized by the nicking endonuclease Nt.BspQI. The horizontal orange lines correspond to the duplicated blocks.

Previously, we attempted to reveal the copy number of *TinCYP51B* genes duplicated in tandem in the genomes of four azole-resistant *T. indotineae* strains TIMM20118, TIMM20119, TIMM20121, and TIMM20122 by combining PacBio long-read sequencing technology with qPCR analysis of gDNA (6). As a result, it was estimated that between 5 and 9 copies of *TinCYP51B* were present in the type I strains TIMM20118 and TIMM20119, respectively. On another side, we estimated the number of *TinCYP51B* at 8 and 14 in strains TIMM20121 and TIMM20122, respectively, both of which are type II and contain 7,374 bp blocks harboring a single *TinCYP51B* gene. In the present study, we performed OGM on HMW gDNA isolated from protoplasts prepared from the growing mycelium of these four resistant strains, as well as from the *T. indotineae* reference strain (IFM66168), which is of type I. OGM successfully revealed the structure of each *TinCYP51B* locus of the five azole-resistant strains (Fig. 1; Fig. S1) and led to the identification of the exact copy number of tandemly duplicated *TinCYP51B* genes in their genomes (Table 2). Both the type I azole-resistant strains TIMM20118 and TIMM20119 were found to have eight copies of *TinCYP51B*. These values were relatively close to the copy numbers previously estimated by PacBio sequencing and qPCR analysis (between 5 and 9 copies, respectively). Eleven copies of *TinCYP51B* were shown in the type I resistant strain IFM66168. In contrast, 19 and 20 copies, respectively, of the type II duplicated blocks harboring the *TinCYP51B* gene were found at each *TinCYP51B* locus of the type II azole-resistant strains TIMM20121 and TIMM20122. These numbers were significantly higher than those estimated by PacBio sequencing and qPCR analysis (10 and 14, respectively) and were approximately twice the number of *TinCYP51B* determined in the three type I resistant strains.

**TABLE 2:** The number of the *TinCYP51B* duplicated blocks in six *T. indotineae* strains revealed by the OGM analysis

*T. indotineae* strains	No. of the *TinCYP51B* duplicated blocks	Strain type
TIMM20114	1	1 *TinCYP51B* gene
IFM66168	11	I
TIMM20118	8	I
TIMM20119	8	I
TIMM20121	19	II
TIMM20122	20	II

### Type I azole-resistant strains exhibit significantly higher *TinCYP51B* expression per gene compared to type II azole-resistant strains

**Fig 2:**
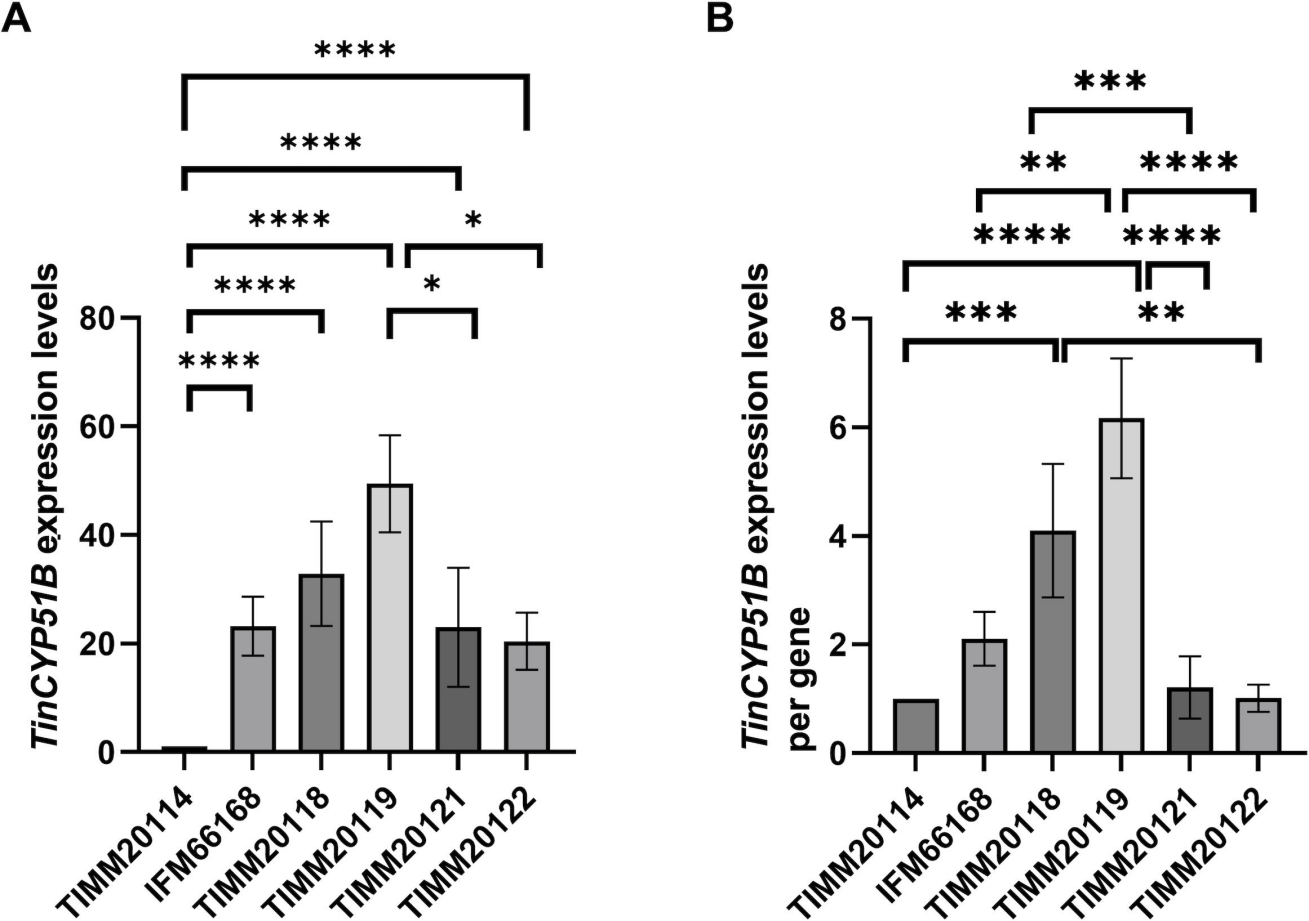
Expression levels of the *TinCYP51B* gene in six *T. indotineae* strains, as determined by qRT-PCR (**A**) and *TinCYP51B* expression levels per gene (**B**). The fold change represents the level of gene expression compared to that of *T. indotineae* TIMM20114. The bars represent the standard deviation of the data obtained from three independent experiments. *P < 0.05; **P < 0.01; ***P < 0.001; ****P < 0.0001.

OGM revealed that the number of copies of the *TinCYP51B* gene in the genomes of type II strains was higher than in type I strains. Conversely, when the expression levels of *TinCYP51B* were compared between type I and type II strains, the average expression level in type I strains was comparable to or higher than that in type II strains (Fig. 2A; Table 3). Thus, the expression level of *TinCYP51B* per gene was significantly higher in the type I strains than in the type II strains (Fig. 2B; Table 3). Also, the expression level of *TinCYP51B* per gene in the type II resistant strains was equivalent to that in the azole-susceptible strain TIMM20114 (Fig. 2B).

**TABLE 3:** Expression of *TinCYP51B* in six *T. indotineae* strains

*T. indotineae* strains	Fold expression of *TinCYP51B* (mean ± SD)^*a*^	Fold expression of *TinCYP51B* per copy no. (mean ± SD)^*b*^	Strain type
TIMM20114	1.0	1.0	1 *TinCYP51B* gene
IFM66168	23.2 ± 4.4	2.1 ± 0.4	I
TIMM20118	32.8 ± 7.8	4.1 ± 1.0	I
TIMM20119	49.4 ± 7.3	6.1 ± 0.9	I
TIMM20121	23.6 ± 8.4	1.2 ± 0.4	II
TIMM20122	22.0 ± 4.3	1.1 ± 0.2	II

^*a*^ Results represent expression levels from three independent real-time PCR experiments. Expression levels of *TinCYP51B* genes were indicated as relative fold changes compared to the ΔCt mean of the data from TIMM20114 (control with a single copy of *TinCYP51B*). SD, standard deviation.

^*b*^ Values indicate the fold expression of *TinCYP51B* gene in each *T. indotineae* strain divided by the number of the *TinCYP51B* duplicated blocks revealed by the OGM analysis. SD, standard deviation.

### Type I azole-resistant strains exhibit both monocistronic and polycistronic transcription of the *TinCYP51B* gene

**Fig 3:**
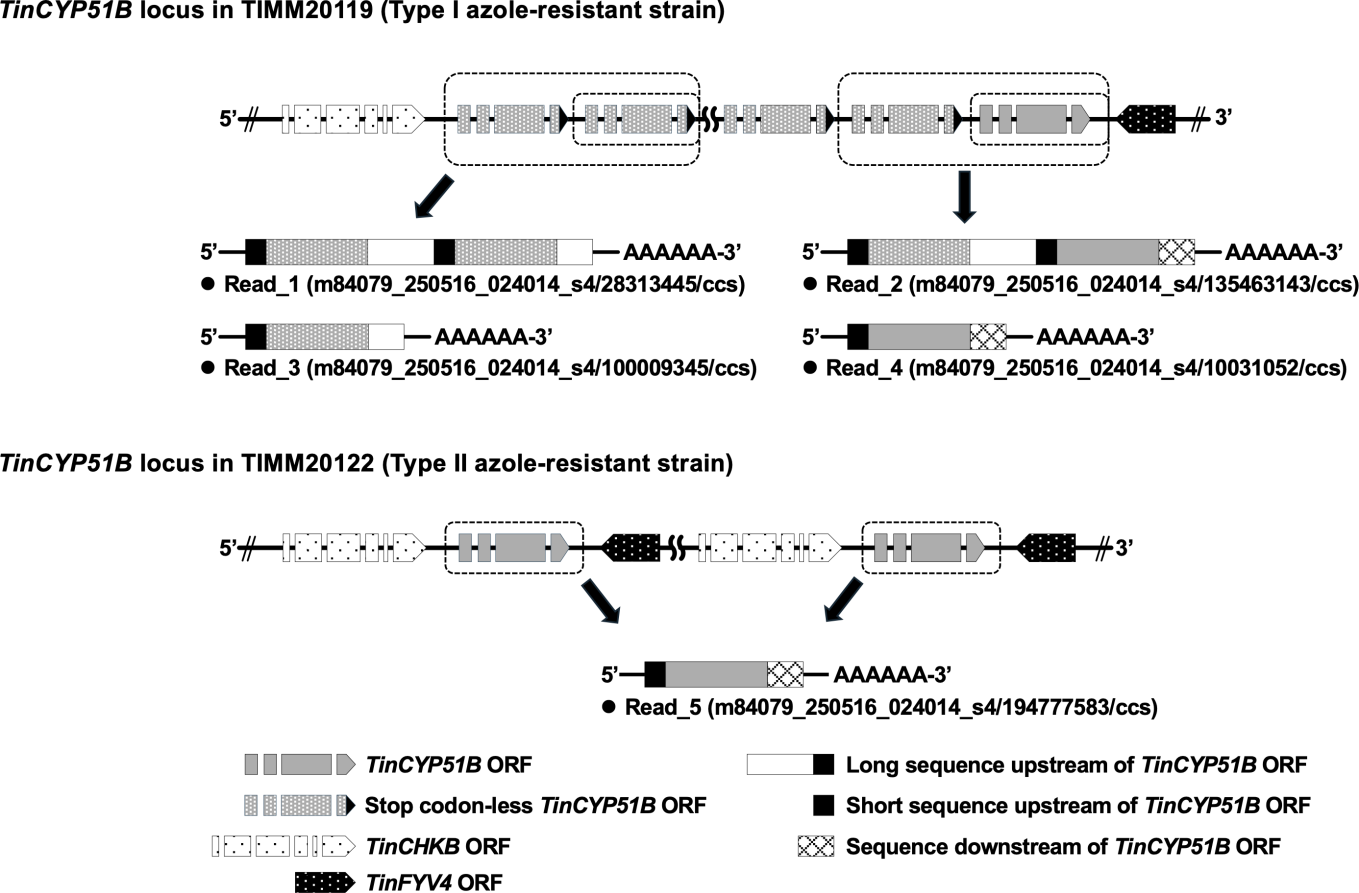
Messenger RNAs with *TinCYP51B* transcripts in the *T. indotineae* type I and type II strains. The sequences were obtained by PacBio Iso-seq RNA long-read sequencing. The reads, including two stop codon-less *TinCYP51B* cDNA sequences or a stop codon-less *TinCYP51B* cDNA sequence and a complete *TinCYP51B* cDNA sequence (reads 1 and 2), together with the reads including a *TinCYP51B* cDNA sequence (reads 3 and 4), were obtained in TIMM20119. Only the reads including a *TinCYP51B* cDNA sequence (read 5) were obtained in the type II azole-resistant strain TIMM20122. The sequences of these five reads are given in the Supplemental material.

As shown in Fig. 1, type II azole-resistant strains harbor *TinCYP51B* tandem duplications consisting of 7,374 bp large blocks, each of which contains *TinCYP51B* as well as the adjacent *TinCHBK* and *TinFYV4* genes, respectively (6). By contrast, the *TinCYP51B* duplications in the type I resistant strains consist of direct tandem repeats of 100% identical blocks of 2,404 bp, inserted at the 5'′ end of the original *TinCYP51B* gene, each of which includes 631 bp of the original promoter and an almost intact coding sequence (CDS) lacking the last five codons, including a stop codon (5). Meanwhile, the blocks do not include the sequence downstream of the *TinCYP51B* CDS required for efficient attenuation of transcription by RNA polymerase II. We hypothesized that the absence of the sequences leading to the termination of transcription between the duplicated *TinCYP51B* CDSs enables the simultaneous transcription of multiple *TinCYP51B* genes under the control of a single promoter. This might explain why *TinCYP51B* expression levels in type I resistant strains are higher than those in type II resistant strains. Therefore, we carried out sequencing of the full-length transcripts in the type I azole-resistant strain TIMM20119 and the type II resistant strain TIMM20122 using the PacBio Iso-seq RNA long-read sequencing method (Table S4). PacBio RNA sequencing provided four types of cDNA reads, including the *TinCYP51B* CDS and polyadenylation [poly (A)] signals in TIMM20119 (Fig. 3): (i) reads starting with the short 5′-upstream sequence and *TinCYP51B* CDS lacking the last five codons, followed by the *TinCYP51B* CDS lacking the last five codons with the long 5′-upstream sequence and ending with a part of the long 5′-upstream sequence; (ii) reads starting with the short 5′-upstream sequence and *TinCYP51B* CDS lacking the last five codons, followed by the intact *TinCYP51B* CDS with the long 5′-upstream sequence and ending with the 3′-downstream sequence; (iii) reads starting with the short 5′-upstream sequence and *TinCYP51B* CDS lacking the last five codons and ending with a part of the long 5′-upstream sequence; and (iv) reads starting with the short 5′-upstream sequence and intact *TinCYP51B* CDS and ending with the 3′-downstream sequence. Furthermore, a region of the long 5′-upstream sequence located in the center of reads, such as reads 1 and 2, including two *TinCYP51B* CDSs, was recognized as an intron (70 bp) and removed by RNA splicing (the region recognized as an intron is shown in the File S1). These results revealed that type I azole-resistant strains exhibit polycistronic transcription of the *TinCYP51B* gene, together with the monocistronic transcription, whereby multiple *TinCYP51B* genes are transcribed into a single molecule of mRNA by a single promoter. Conversely, for TIMM20122, only reads including a single intact *TinCYP51B* CDS were found, such as for the azole-susceptible strains TIMM20114 and TIMM20115. Sequences of the five reads (reads 1 to 5) shown in Fig. 3 are given in the File S1 .

### OGM revealed an approximately 970 kb large genomic DNA inversion located downstream of the *TinCYP51B* locus in type I azole-resistant *T. indotineae* strains

**Fig 4:**
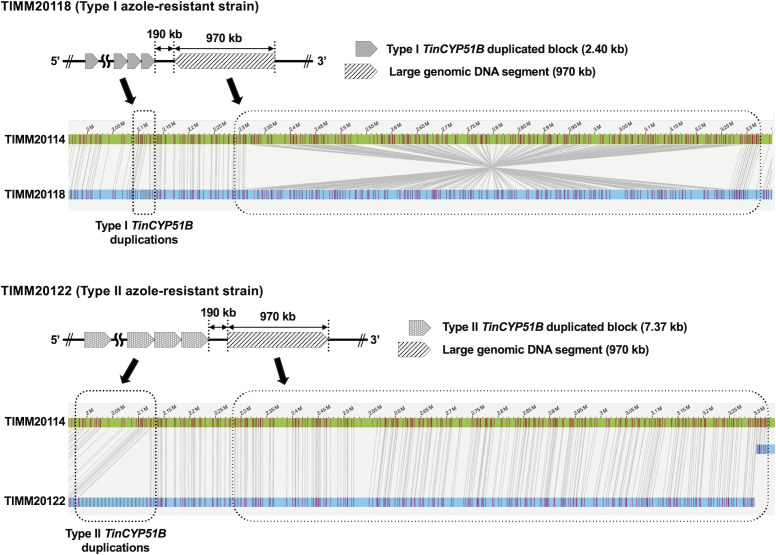
Comparison of genomic maps of TIMM20114, TIMM20118, and TIMM20122. OGM results revealed the inversion of a large fragment of approximately 970 kb on chromosome 3 (the contig 2, ctg.000002F) of the azole-resistant strain TIMM20118 compared to TIMM20114. This fragment is located approximately 190 kb downstream of the *TinCYP51B* locus. By contrast, the orientation of this fragment is the same in both TIMM20122 and TIMM20114.

As mentioned above, OGM successfully revealed the structure of each *TinCYP51B* locus in the genomes of six *T. indotineae* strains (Table 2) and led to the identification of the exact copy number of tandemly duplicated *TinCYP51B* genes in their genomes (Table 2). Meanwhile, a comparison of the OGM data from the types I and II azole-resistant strains revealed the presence of an approximately 970 kb gDNA segment oriented in the opposite direction, located about 190 kb downstream of their *TinCYP51B* loci (Fig. 4; Fig. S2). The orientation of the large gDNA segment in the two type II resistant strains, TIMM20121 and TIMM20122, was identical to that in the azole-susceptible strain, TIMM20114, used as a reference genome for OGM analysis, suggesting that the gDNA region in the type Iresistant strains was inverted.

**Fig 5:**
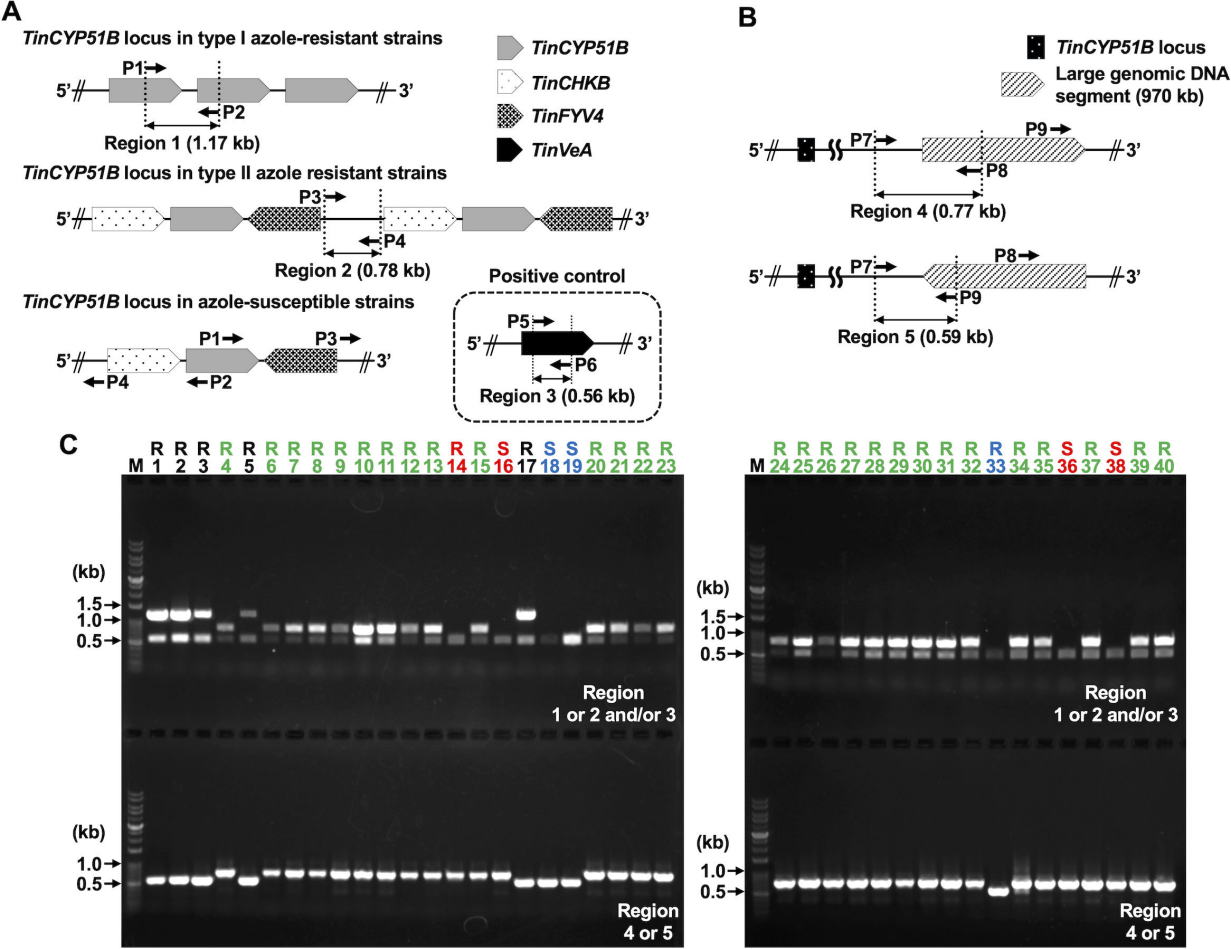
Rapid identification of azole-resistant *T. indotineae* strains harboring the type I or II *TinCYP51B* tandem sequences and detection of the about 970 kb large genomic inversion located downstream of the *TinCYP51B* locus. A search was conducted on the *T. indotineae* strains listed in Table S1. (**A**) Identification of azole-resistant strains harboring the type I or II *TinCYP51B* tandem sequences by multiplex PCR amplification of gDNA. Three sets of primers were designed: (i) P1-P2 for the type I *TinCYP51B* tandem sequence; (ii) P3-P4 for the type II *TinCYP51B* tandem sequence; and (iii) P5-P6 for an internal fragment of the *TinVeA* gene (a homolog of *Aspergillus nidulans veA*, accession no. ANIA_01052) as a positive control, respectively. (**B**) Detection of the large inverted genomic segment by multiplex PCR amplification of *T. indotineae* gDNA using a set of P7-P8-P9 primers. (**C**) Separation of amplicons by agarose gel electrophoresis to identify type I and type II strains and detect the orientation of the large 970 kb genomic region. Top gel, fragments of 1.17 kb, 0.78 kb, and 0.56 kb were amplified with primer pairs P1-P2, P3-P4, and P5-P6, respectively. The 1.17 kb and 0.78 kb fragments are specific to type I and type II strains, respectively. The 0.56 kb fragment of *TinVeA* is a positive control detected in all strains of *T. indotineae*. Bottom gel, fragments of 0.77 kb and 0.59 kb were amplified with primers P7-P8 and P7-P9, respectively. A 0.77 kb fragment confirms the orientation of the large 970 kb genomic segment as in TIMM20114, while a 0.59 kb fragment confirms the orientation of the large 970 kb genomic segment as in TIMM20115. The lane number for each sample corresponds to the strain number listed in Table S1. The strains corresponding to the black lane number have the type I *TinCYP51B* tandem sequence together with the large inverted genomic segment, while the strains corresponding to the green lane number have the type II *TinCYP51B* tandem sequence. Strains with a red track number have the large genomic segment in the same orientation as strain TIMM20114. They are susceptible strains with one copy of the *TinCYP51B* gene or resistant strains carrying neither type I nor type II *TinCYP51B* tandem sequence. Strains with a blue track number have a large genomic segment inverted relative to TIMM20114, like TIMM20115. TIMM20114 (lane 16), TIMM20119 (lane 2), and TIMM20122 (lane 10) were used as positive controls for a wild-type strain harboring one copy of the *TinCYP51B* gene, and azole-resistant strains harboring the type I or II *TinCYP51B* tandem sequence, respectively. The letter R indicates azole resistance, while the letter S indicates azole susceptibility. M, Molecular weight marker.

Subsequently, we attempted to identify the azole-resistant strains harboring the type I or II *TinCYP51B* tandem sequences and detect the 970 kb large gDNA inversion located downstream of the *TinCYP51B* locus in other *T. indotineae* strains listed in Table S1 by using multiplex PCR amplification with their gDNA samples. Three pairs of PCR primers were designed for rapid identification of the type I and type II strains (Fig. 5A): P1-P2 for the type I *TinCYP51B* tandem sequence, P3-P4 for the type II *TinCYP51B* tandem sequence, and P5-P6 for an internal fragment of the *TinVeA* gene (a homolog of the *Aspergillus nidulans veA* gene) as a positive control (Fig. 5A). To detect the large genomic inversions, primers P7, P8, and P9 were designed (Fig. 5B). Amplification of an about 0.77 kb fragment shows the orientation of the large 970 kb gDNA segment as that in TIMM20114, while an approximately 0.59 kb DNA fragment would be amplified when the large inverted gDNA segment was present. Our results showed that the large gDNA segment in all type II azole-resistant strains had the same orientation as TIMM20114. In contrast, the orientation of this gDNA segment in all the type I resistant strains was opposite to that of TIMM20114 (Fig. 5C; Table S1). The two azole-susceptible strains, TIMM20115, which had been revealed to harbor a single *TinCYP51B* gene by our previous WGS (5), and UKJ 1691/17 showed that the large gDNA segment was oriented in the opposite direction to that of TIMM20114 (lanes 18 and 19 in Fig. 5C).

Four strains of *T. indotineae* with reduced azole susceptibility, 250150/18, 600098/19, 600113/19, and 600126/19 (lanes 14, 33, 36, and 38, respectively, in Fig. 5C), were of neither type I nor type II. The MIC data were corroborated by Etests and serial dilution drug susceptibility assays, which revealed that these strains exhibited significantly lower ITC and VRC susceptibilities than TIMM20114 (Fig. S3). qRT-PCR analysis revealed that the expression level of the *TinCYP51B* gene was about nine-, seven-, and threefold higher in strains 250150/18, 600098/19, and 600113/19, respectively, than in strain TIMM20114 (Table S5). Furthermore, the expression level of the *TinCYP51B* gene in strain 600126/19 was equivalent to that in TIMM20114. To estimate the copy number of the *TinCYP51B* gene in the genomes of these four strains, we performed a Southern blotting analysis of their gDNAs digested with the XhoI restriction enzyme (Fig. S4). This analysis detected a *TinCYP51B* band, with a signal intensity similar to that of the susceptible strain TIMM20114, in the three strains 600098/19, 600113/19, and 600126/19 (Fig. S4, lanes 5 to 7), suggesting that these three strains harbor a single *TinCYP51B* gene. Strain 250150/18 also showed one *TinCYP51B* band (Fig. S4, lane 4), but its signal intensity was significantly higher than that of the other three strains with reduced azole susceptibility and TIMM20114, indicating the presence of multiple *TinCYP51B* copies. These results suggest that azole resistance in *T. indotineae* may be due to mechanisms different from those in types I and II strains. The orientation of the large gDNA segment in strain 600098/19 was identical to that in TIMM20115. In contrast, the orientation of the large gDNA segment in the strains 250,150/18, 600,113/19, and 600,126/19 was identical to that in TIMM20114 (Fig. 5C; Table S1).

## DISCUSSION

OGM analysis enabled us to visualize the entire structure of the *TinCYP51B* locus, including the *TinCYP51B* tandem duplications, thereby determining the precise copy number of the *TinCYP51B* gene in the *T. indotineae* genomes. In addition, OGM revealed structural characteristics in the genomes of the *T. indotineae* strains, providing clues regarding how to elucidate the evolutionary process of its resistance to azoles.

### Different resistance mechanisms in the type I and type II strains

Successfully determining the exact copy number of the *TinCYP51B* gene revealed an inverse relationship between its copy number and expression levels in the types I and II azole-resistant strains. The copy number of the *TinCYP51B* gene in the type I resistant strains was significantly lower than in the type II resistant strains (Table 2), while the *TinCYP51B* expression level per gene in the type I resistant strains was significantly higher than in the type II resistant strains (Fig. 2B; Table 3). This indicates that the type I *TinCYP51B* tandem duplications are more efficient at expressing the *TinCYP51B* gene than type II tandem duplications. Consequently, type I resistant strains can produce sterol demethylase more efficiently than type II resistant strains, giving them a greater advantage in expressing azole resistance.

**Fig 6:**
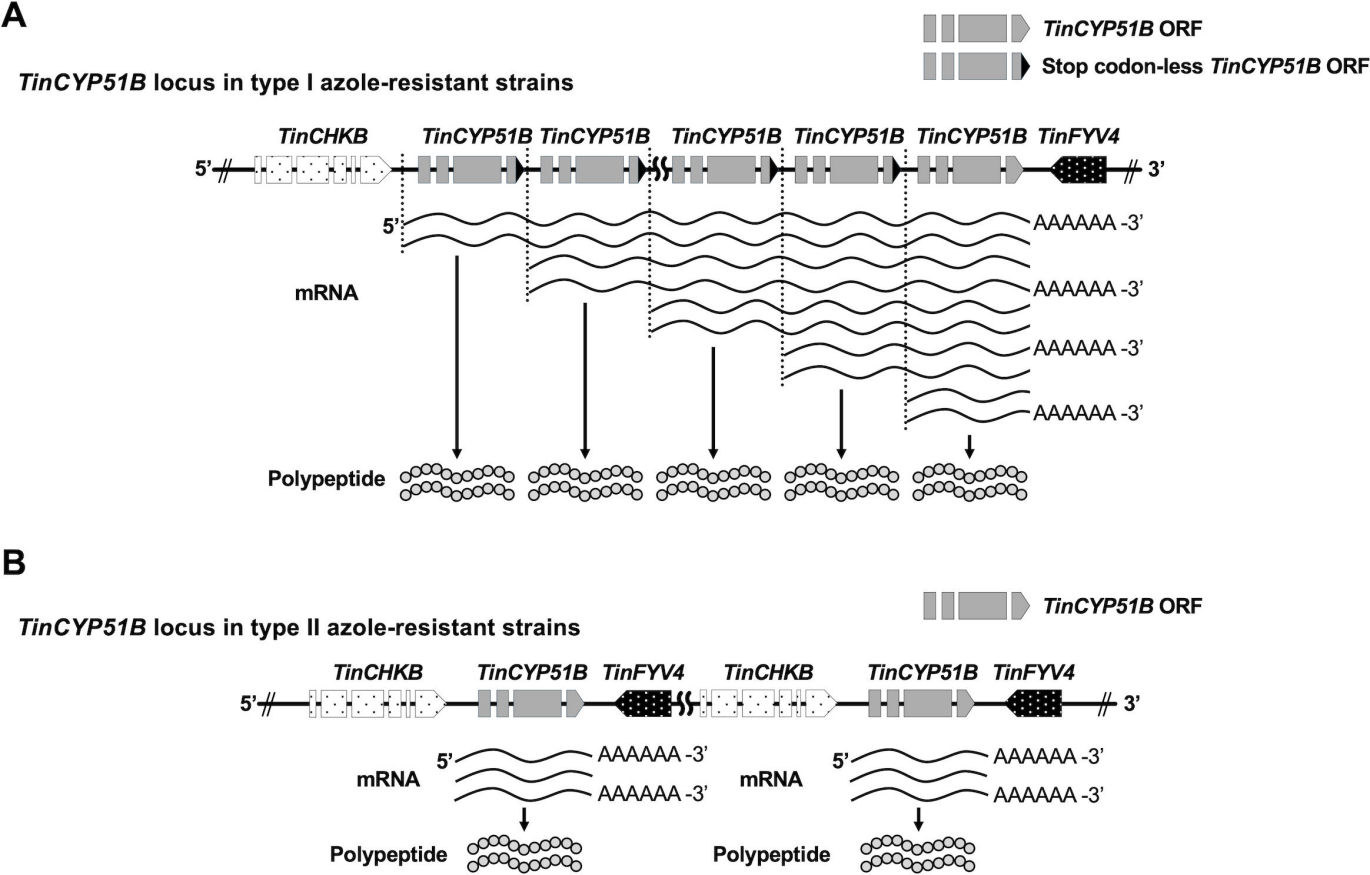
Comparison of the transcriptional landscapes in the *TinCYP51B* locus between the type I and II azole-resistant *T. indotineae* strains. (**A**) Single mRNA (polycistronic mRNA) molecules that carry genetic information for multiple *TinCYP51B* genes, together with mRNA (monocistronic mRNA) molecules that encode just a single CYP51B polypeptide, are transcribed in the *TinCYP51B* locus of the type I azole-resistant strains. (**B**) Only the monocistronic mRNA molecules are transcribed in the *TinCYP51B* locus of the type II azole-resistant strains.

Structural differences in duplicated blocks between the *TinCYP51B* loci of the type I and type II strains can explain the different levels of *TinCYP51B* expression. In type II strains harboring large segmental duplications, each *TinCYP51B* ORF is located between the promoter and the downstream transcription attenuation sequence. Thus, transcriptional machinery generates typical monocistronic mRNAs, including one *TinCYP51B* CDS from each duplication block. On the other hand, the tandem duplications of the type I strains consist of 2,404 bp blocks, each block containing the *TinCYP51B* ORF and its promoter but lacking the transcription attenuation sequence. Since there is the transcription attenuation sequence only downstream of the most 3'′ located *TinCYP51B* ORF throughout the entire tandem duplications, multiple *TinCYP51B* ORFs are transcribed into a single mRNA. When the copy number is 8, as in TIMM20119, mRNAs including eight *TinCYP51B* CDSs are theoretically generated from the first promoter, mRNAs including seven *TinCYP51B* CDSs are generated from the second promoter, mRNAs including six *TinCYP51B* CDSs are generated from the third promoter, and so on, as explained in Fig. 6. In the present study, dicistronic mRNAs including two *TinCYP51B* CDSs were found by the PacBio Iso-seq RNA long-read sequencing method, indicating that transcription of the polycistronic mRNAs containing multiple *TinCYP51B* CDSs is, indeed, occurring in the type I resistant strains. Polycistronic gene expression, which is commonly observed in prokaryotes, was once thought to be extremely rare in eukaryotes. Nevertheless, this is not the case for organelles of prokaryotic origin, such as mitochondria and chloroplasts, which have extremely compact genomes and express many genes on polycistronic transcripts. However, recent advances in sequencing technologies have revealed that, alongside monocistronic mRNA and its isoforms, many read-through transcripts, including polycistronic mRNA, are present in some eukaryotes, including mushroom-forming fungi (17–20). In *T. indotineae* type I strains, polycistronic transcription enhances the impact of the *TinCYP51B* duplication on azole resistance production, with a greater overexpression of the gene that encodes the drug target.

Transcripts with a molecular weight of 5 kb or higher, which contained three or more *TinCYP51B* CDSs, were not detected in our analysis. Technical limitations related to total RNA extraction and conversion of RNA to cDNA may explain this result: first, longer mRNA molecules are more vulnerable to fragmentation during standard total RNA extraction processes due to multiple factors, including mechanical, chemical, and enzymatic degradation. Second, the conversion of mRNA to cDNA can be inefficient for very long transcripts, resulting in the generation of truncated cDNA molecules. Third, during PCR library preparation for sequencing, shorter cDNA fragments were preferentially amplified, resulting in reduced amplification of cDNA fragments derived from long transcripts. Alternatively, transcripts with a molecular weight of 5 kb or higher are scarcely synthesized in the fungal cells, with only dicistronic mRNA encoding two TinCYP51B polypeptides being produced.

### A large-scale inversion in genomes revealed by OGM delineates two groups of *T. indotineae* strains

**Fig 7:**
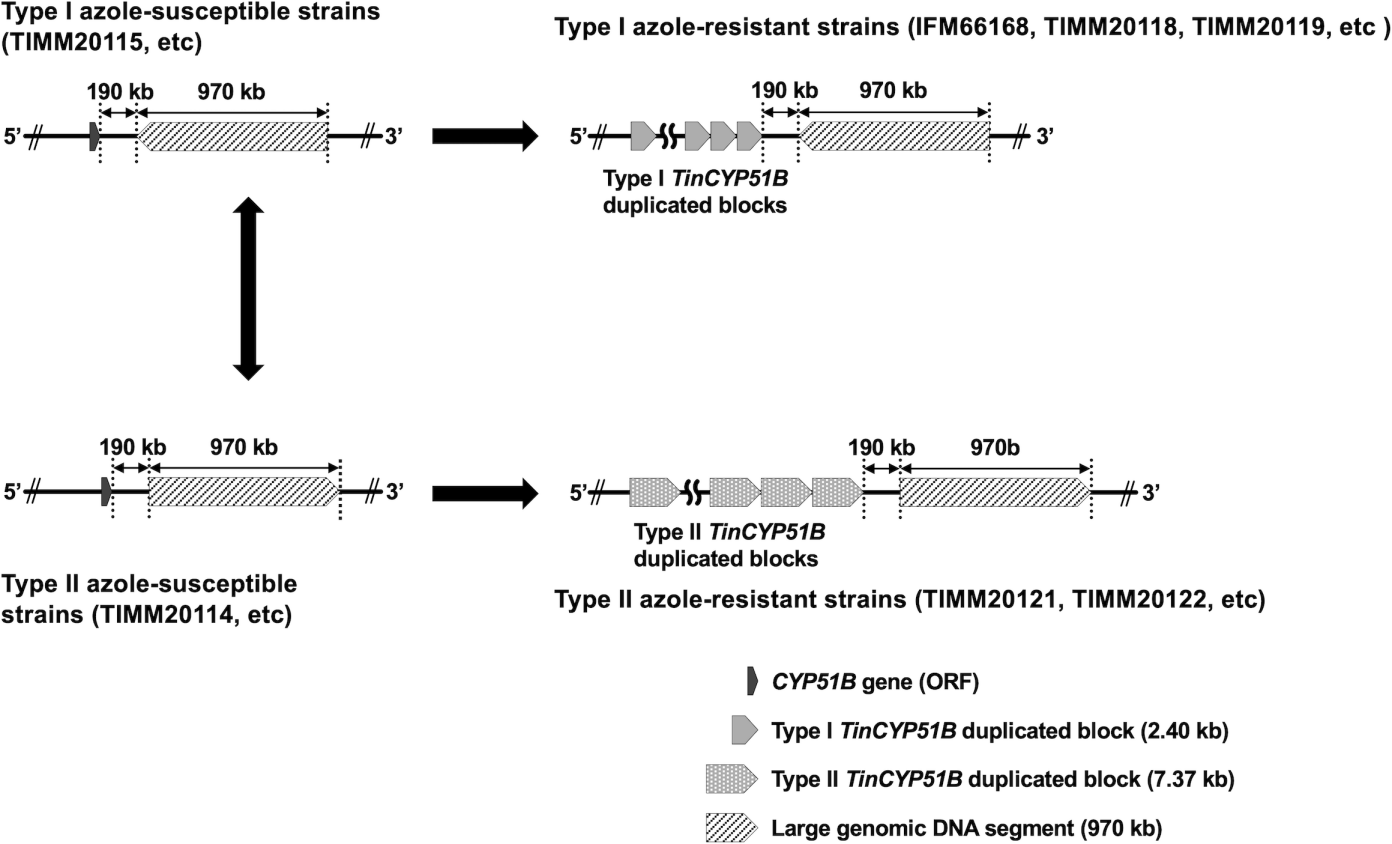
Evolutionary process from azole-susceptible strains to the emergence of two distinct types of azole-resistant strains in *T. indotineae*, under azole stress.

In addition to covering the entire genomes of *T. indotineae* strains, OGM revealed that an approximately 970 kb region on chromosome 3 (the contig 2) was inverted in TIMM20015 and type I azole-resistant strains compared to TIMM20014 and type II resistant strains. Genome SVs revealed the evolutionary process from azole-susceptible strains to the emergence of two distinct types of azole-resistant strains in *T. indotineae*. Based on the genotype and 970 kb gDNA region orientation of over 40 *T. indotineae* strains (Table S1 and recently obtained unpublished strains), as shown in Fig. 7, it is hypothesized that type I azole-resistant strains have arisen from a susceptible strain, such as TIMM20115, that possesses only the *TinCYP51B* gene after exposure to azole stress. Conversely, type II azole-resistant strains are thought to have arisen from a susceptible strain such as TIMM20114, which also has only one *TinCYP51B* gene, that was subjected to azole stress. A genetic event resulted in an inversion of the 970 kb gDNA region in a susceptible strain, forming a new strain with an inverted region orientation. These results support the idea that type I and type II strains belong to two distinct clusters, which is consistent with the findings by Xie et al. (21). Noteworthy, through a recent survey of *T. indotineae* based on WGS data of 180 strains from around the world, Xie et al. (21) revealed that all the type I strains were from China. In contrast, most azole-resistant strains isolated in India (1) were of type II. Given the above findings, the orientation of the 970 kb gDNA region appears to be evidently correlated with the geographic distribution of two populations existing within one species of organism, *T. indotineae*. Chromosomal inversions, which involve the reversal of chromosome segments by 180° without significant gain or loss of genetic materials, have been considered crucial contributors to the evolutionary process in diverse organisms (22–28). Accordingly, inversion of the 970 kb gDNA region provides a crucial genetic marker for understanding the evolutionary trajectory of T. *indotineae* and how the two types of azole-resistant strains arose and spread.

### Other types of resistance mechanisms in *T. indotineae*

Of the 40 *T. indotineae* strains investigated in the present study, 37 were resistant to azole compounds. Thirty-three of these strains were either type I or type II. The remaining four strains with reduced azole susceptibility, however, did not fall into either category. Southern blotting (Fig. S4) suggested that strain 250,150/18 would belong to a different type containing multiple *TinCYP51B* gene copies. No further investigations were conducted on this strain in our laboratory. However, in connection with this, the existence of a new type III resistance strain containing tandem sequences of approximately 21 kb, including the *TinCYP51B* gene, has been reported (21). In contrast, strains 600,098/19, 600,113/19, and 600,126/19 appeared to have only one *TinCYP51B* gene. In summary, these four strains are believed to possess alternative resistance mechanisms to azole compounds that are not associated with either type I or type II tandem repeat sequences.

### Prospects for studying resistance in organisms other than *T. indotineae*

Amplification of the genes encoding the target molecules of toxic chemicals is not restricted to *T. indotineae*; it also occurs in other fungi, plants, insects, and bacteria (6). Generally, reports of gene duplications have indicated large-scale segmental duplications in the genome. This occurred in the genomes of glyphosate-resistant weeds, in which duplications were detected by qPCR with gDNA as a target and fluorescence *in situ* hybridization (FISH) (29). The same was true for the genomes of antifungal-resistant isolates of a plant pathogen, *Rhychosporium commune* (30), and the genome of the DDT-resistant mosquito *Anopheles stephensi* (which transmits the malaria parasite) (31). These were revealed by Illumina next-generation sequencing technology. Sequences with multiple copies of the same genes in tandem, each with its own promoter, but the transcription attenuation sequence only lying downstream of the most 3′ located *TinCYP51B* ORF throughout the entire tandem duplications, have only been found in type I strains of *T. indotineae*. In any event, *de novo* PacBio assemblies are generally insufficient for generating nearly complete, gap-free genomes without optical mapping (32, 33). The entire locus conferring resistance to azole compounds in *T. indotineae* could be sequenced using PacBio sequencing technology coupled with OGM. The Saphyr system from Bionano, coupled with long-read sequencing technology such as PacBio, is a method of choice for analyzing loci involved in resistance when the genes targeted by an antifungal or toxic compound are repeated in tandem.

## MATERIALS AND METHODS

### Strains and growth media

Strains of *T. indotineae* (*N* = 40), which had been previously characterized for susceptibility to ITC and VRC (1), were used in this study (Table S1). Of these strains, 37 were deemed to be resistant to ITC and VRC (ITC MIC_80_ ≥ 0.25 µg/mL and VRC MIC_80_ ≥ 0.25 µg/mL). The cut-off point was determined based on the abnormal distribution of MIC values for VRC and ITC across a wide range of *T. indotineae* strains (1). For VRC, two distinct peaks of MIC frequencies were observed, one for MICs higher than 0.25 µg/mL, and the second for MICs lower than 0.25 µg/mL. The two previously characterized strains, TIMM20114 and TIMM20115, which harbor only one *TinCYP51B* gene, were used as azole-susceptible controls (ITC MIC_80_ = 0.06 µg/mL, and VRC MIC_80_ = 0.015 µg/mL and 0.03 μg/mL, respectively). The strains selected for OGM, and the study of the *TinCYP51B* gene expression mechanisms are listed in Table 1.

Sabouraud dextrose agar (SDA) and Sabouraud dextrose broth (SDB) (BD Bioscience) were used for the conventional culture of these strains. Spore formation was promoted at 28°C using 1/10 SDA (a 10-fold dilution of SDB, 1.5% [wt/vol] Bacto agar [BD Bioscience]) supplemented with 500 µg/mL cycloheximide and 50 µg/mL chloramphenicol (Fujifilm Wako Pure Chemical Corporation). The MICs were measured as previously described (34).

### Optical genome mapping (OGM) and data analysis

The OGM and data analysis of the five *T. indotineae* strains IFM66168, TIMM20118, TIMM20119, TIMM20121, and TIMM20122 (Table 1) were performed by AS ONE CORPORATION (Japan). The high molecular weight (HMW) gDNA was isolated using the NucleoBond HMW DNA kit (MACHEREY-NAGEL GmbH & Co. KG) from protoplasts, which were prepared from the growing mycelia according to a method described by Yamada et al. (35). The OGM was performed on the Saphyr platform (Bionano Genomics). In short, HMW gDNA was fluorescently labeled using a Bionano Prep NLRS labeling kit (for TIMM20118, TIMM20119, TIMM20121, and TIMM20122) or a Bionano Prep DLS-G2 labeling kit (for IFM66168), followed by automated electrophoresis in the nanochannel array of the Saphyr Chip and automated sequential imaging of the linearized DNA, achieved by using the Bionano Saphyr instrument. The cumulative length of DNA molecules that are 150 kb or longer (counts toward the throughput target) was set at 50 Gb for data collection for each sample. Molecular statistical data indicating the quality of the samples analyzed are summarized in Table S2. The average filtered N50 (≥ 150 kb) molecule length was 200 kb. Label density was ≥ 9.7/100 kb. An effective coverage of the reference genome with > 500× was achieved for all gDNA samples.

Data processing was performed using Bionano Solve software (ver. 3.8.2.1), and the OGM-specific pipelines were managed via Bionano Access software (ver. 1.8.2.1). A *de novo* assembly pipeline was used for the identification of SVs, including copy number variants (CNVs). The CNVs of the *TinCYP51B* gene duplicated block were detected based on alignment between the *de novo* assembled genome maps and the whole-genome sequence (consisting of three contigs) of *T. indotineae* strain TIMM20114 (accession no. JAJVHL000000000) (5), used as a reference genome. In the present study, the three contigs were designated as chromosome 1 (the contig 0, ctg.000000F), chromosome 2 (the contig 1, ctg.000001F), and chromosome 3 (the contig 2, ctg.000002F), respectively.

### Rapid PCR identification of type I and II azole-resistant strains of *T. indotineae*

For rapid strain typing of azole-resistant *T. indotineae* by multiplex PCR, gDNA was extracted from the growing mycelia according to a method described by Girardin et al. (36). Aliquots of 30 ng of the gDNA were used as templates for multiplex PCR with three sets of primers: P1-P2 for type I azole-resistant strains, P3-P4 for type II azole-resistant strains, and P5-P6 for amplification of the *TinVeA* gene (a homolog of *Aspergillus nidulans veA*, accession no. ANIA_01052), which was used as a positive control (Fig. 5A). The nucleotide sequence of the primers used for multiplex PCR is listed in Table S3. The PCR reaction was carried out in a 25 µL reaction mixture containing 30 ng of gDNA, 0.6 U PrimeSTAR HS DNA polymerase (Takara Bio), and 0.2 µM primers. The PCR cycling conditions consisted of initial denaturation at 98°C for 1 min, followed by 25 cycles at 98°C for 10 sec, annealing at 70°C for 15 sec, and extension at 72°C for 1.2 min. The PCR products were electrophoresed on 0.8% (wt/vol) agarose gels and visualized under the gel documentation system.

### Real-time quantitative PCR

For the relative quantification of the *TinCYP51B* gene expression in *T. indotineae* strains by real-time quantitative reverse transcription PCR (qRT-PCR), total RNA extraction from the growing mycelia and first-strand cDNA synthesis were performed, as described previously for *T. rubrum* (37). The synthesized cDNAs were treated with DNase I (Qiagen) before use. qRT-PCR was performed using the Power SYBR green PCR master mix on a StepOne real-time PCR system (Thermo Fischer Scientific), under standard conditions, according to the manufacturer’s recommendations. The 2^−ΔΔCt^ method was used to compare the ΔCt (threshold cycle [Ct] of the target gene [*TinCYP51B*] minus the Ct of the endogenous control gene [actin gene, *TinACT*]) values of the test strains (with unknown target gene copy numbers) with the ΔCt value of a calibrator strain (TIMM20114), which had a single copy target gene. The glyceraldehyde-3-phosphate dehydrogenase gene (*TinGAPDH*) was added as a target gene to confirm that there was virtually no difference in PCR reaction efficiency among the analyzed strains. The primers used to amplify *TinCYP51B*, *TinGAPDH*, and *TinACT* are listed in Table S3. Dissociation curves of the amplified products were plotted to confirm the absence of nonspecific products or primer dimers.

The statistical significance of the expression levels of target genes among strains was assessed using one-way ANOVA and Tukey’s post hoc test on log10-transformed PCR data (to ensure normality).

### PacBio Iso-seq RNA long-read sequencing

The RNA sequencing and data analysis of *T. indotineae* strains were performed by the Bioengineering Lab. Co., Ltd. (Japan). *Trichophyton indotineae* strains TIMM20119 and TIMM20122 were cultured in 10 mL of SDB in 50-mL conical tubes (Corning) without antifungal drugs at 28°C for 3–5 days. The growing mycelia from each strain were harvested and frozen under liquid nitrogen. Total RNA was extracted from mycelial samples ground with the Multi-Beads Shocker (Yasui Kikai) by using the RNeasy Mini kit (Qiagen). The quantity and quality of extracted total RNA were assessed by using the Quantus Fluorometer/the QuantiFluor RNA system (Promega) and the 5200 Fragment Analyzer System/the Agilent HS RNA Kit (Agilent Technologies). Three hundred nanograms of total RNA was used for cDNA synthesis. First-strand cDNA synthesis and PCR amplification of cDNA products were performed by using the Iso-seq Express 2.0 Kit (Pacific Biosciences), according to the manufacturer’s instructions. The amplified cDNA products were purified using SMRTbell cleanup beads (Pacific Biosciences) before library preparation. The SMRTbell libraries were prepared using 130 ng of the purified cDNA products with the SMRTbell prep kit 3.0 (Pacific Biosciences), according to the manufacturer’s instructions. For binding to a polymerase, the SMRTbell libraries were bound to DNA polymerase molecules by using a Revio Polymerase kit (Pacific Biosciences). The prepared SMRTbell libraries, with bound polymerase molecules, were loaded onto the Revio system (Pacific Biosciences) and sequenced. The HiFi reads were generated using the SMRT Link (ver. 25.2.0.266456) as follows. Adapter sequences were removed from the obtained sequences, resulting in the generation of subreads. After creating circular consensus sequences by aligning the subreads, those with an average quality value (< 20) were removed, and the remaining circular consensus sequences were designated as HiFi reads.

### PCR-based detection of the large inverted genomic segment located downstream of the *TinCYP51B* locus in the *T. indotineae* genome

Orientation of a large chromosomal region (about 970 kb) located downstream of the *TinCYP51B* locus in the *T. indotineae* strains was identified by multiplex PCR with a set of primers: P7, P8, and P9 (Fig. 5B), the nucleotide sequence of which is listed in Table S3. The PCR reaction was carried out in a 20 µL reaction mixture containing 40 ng of gDNA, 0.5 U rTaq DNA polymerase (Takara Bio), and 0.2 µM primers. The PCR cycling conditions consisted of initial denaturation at 94°C for 10 sec, followed by 35 cycles at 94°C for 30 sec, annealing at 60°C for 30 sec, and extension at 72°C for 45 sec. The PCR products were electrophoresed on 0.8% (wt/vol) agarose gels and visualized under the gel documentation system.

## AFTER EPUB

*[This article was published on 31 March 2026 with a typo in Fig. 3. Figure 3 was corrected in the current version, posted on 17 April 2026.]*

## SUPPLEMENTAL MATERIAL

Fig. S1 aac.01817-25-s0001.pdf *TTinCYP51B* gene tandem duplications in TIMM20118 (type I), IFM66168 (type I) and TIMM20121 (type II), visualized by OGM.

Fig. S2 aac.01817-25-s0002.pdf Large gDNA inversion located downstream of the *TinCYP51B* locus in the type I azole-resistant *T. indotineae* strain TIMM20119 and IFM66168, visualized by OGM.

Fig. S3 aac.01817-25-s0003.pdf Susceptibilities to ITC and VRC of the strains 250150/18, 600098/19, 600113/19, and 600126/19 compared to TIMM 20114 (the susceptible strain) and TIMM 20119 and TIMM 2020122 (type I and type II resistant strains, respectively).

Fig. S4 aac.01817-25-s0004.pdf Southern blotting analysis of gDNA samples from T. indotineae strains.

Table S1 aac.01817-25-s0005.pdf Phenotypic and genotypic characteristics of *T. indotineae* strains used in this study.

Table S2 aac.01817-25-s0006.pdf Molecule statistics.

Table S3 aac.01817-25-s0007.pdf Oligonucleotide primers used in this study.

Table S4 aac.01817-25-s0008.pdf Summary of results on sequencing of the full-length transcripts in the two *T. indotineae* strains using PacBio Iso-seq RNA long-read sequencing method.

Table S5 aac.01817-25-s0009.pdf Expression of *TinCYP51B* in *T. indotineae* strains with reduced azole susceptibility.

File S1 aac.01817-25-s0010.pdf The cDNA reads in the type I azole-resistant strain TIMM20119 and the type II-resistant strain TIMM20122.

ASM does not own the copyrights to Supplemental Material that may be linked to, or accessed through, an article. The authors have granted ASM a non-exclusive, world-wide license to publish the Supplemental Material files. Please contact the corresponding author directly for reuse.
