# Supplementary material for: The entire CYP51B locus in azole-resistant isolates of the dermatophyte Trichophyton indotineae revealed by optical genome mapping
Source: Antimicrob Agents Chemother. 2026 Mar 31;70(5):e01817-25. doi: 10.1128/aac.01817-25 (PMC13148020; doi:10.1128/aac.01817-25)
Supplement: Fig. S1 — TTinCYP51B gene tandem duplications in TIMM20118 (type I), IFM66168 (type I) and TIMM20121 (type II), visualized by OGM. [file aac.01817-25-s0001.pdf]

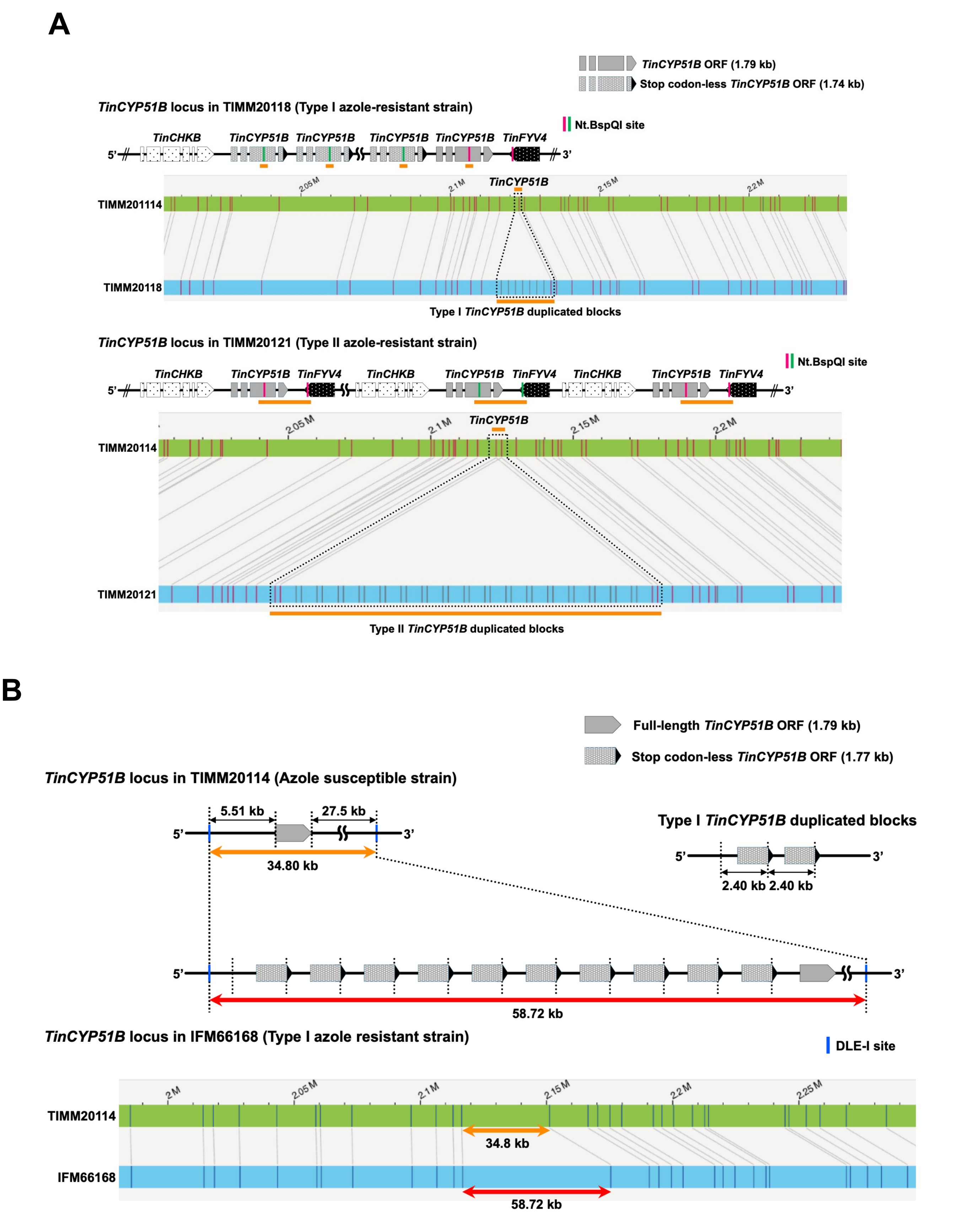

Fig. S1. *TinCYP51B* gene tandem duplications in TIMM20118 (type I), IFM66168 (type I) and TIMM20121 (type II), visualized by OGM. The green bars show chromosome 3 (the contig 2, ctg.000002F) of TIMM20114 used as a reference sequence, and blue bars show the maps assembled from the OGM data of TIMM20118, IFM66168 and TIMM20121. The vertical pink and green lines on the bars in Fig. S1A indicate the location and pattern of the GCTCTTG motif recognized by the nicking endonuclease Nt.BspQI and the vertical blue lines on the bars in Fig. S1B indicate the location and pattern of the CTTAAG motif recognized by the DLE-I enzyme with a methyltransferase-like function. The horizontal orange lines in Fig. S1A correspond to the duplicated blocks.
