# Supplementary material for: The entire CYP51B locus in azole-resistant isolates of the dermatophyte Trichophyton indotineae revealed by optical genome mapping
Source: Antimicrob Agents Chemother. 2026 Mar 31;70(5):e01817-25. doi: 10.1128/aac.01817-25 (PMC13148020; doi:10.1128/aac.01817-25)
Supplement: Fig. S2 — Large gDNA inversion located downstream of the TinCYP51B locus in the type I azole-resistant T. indotineae strain TIMM20119 and IFM66168, visualized by OGM. [file aac.01817-25-s0002.pdf]

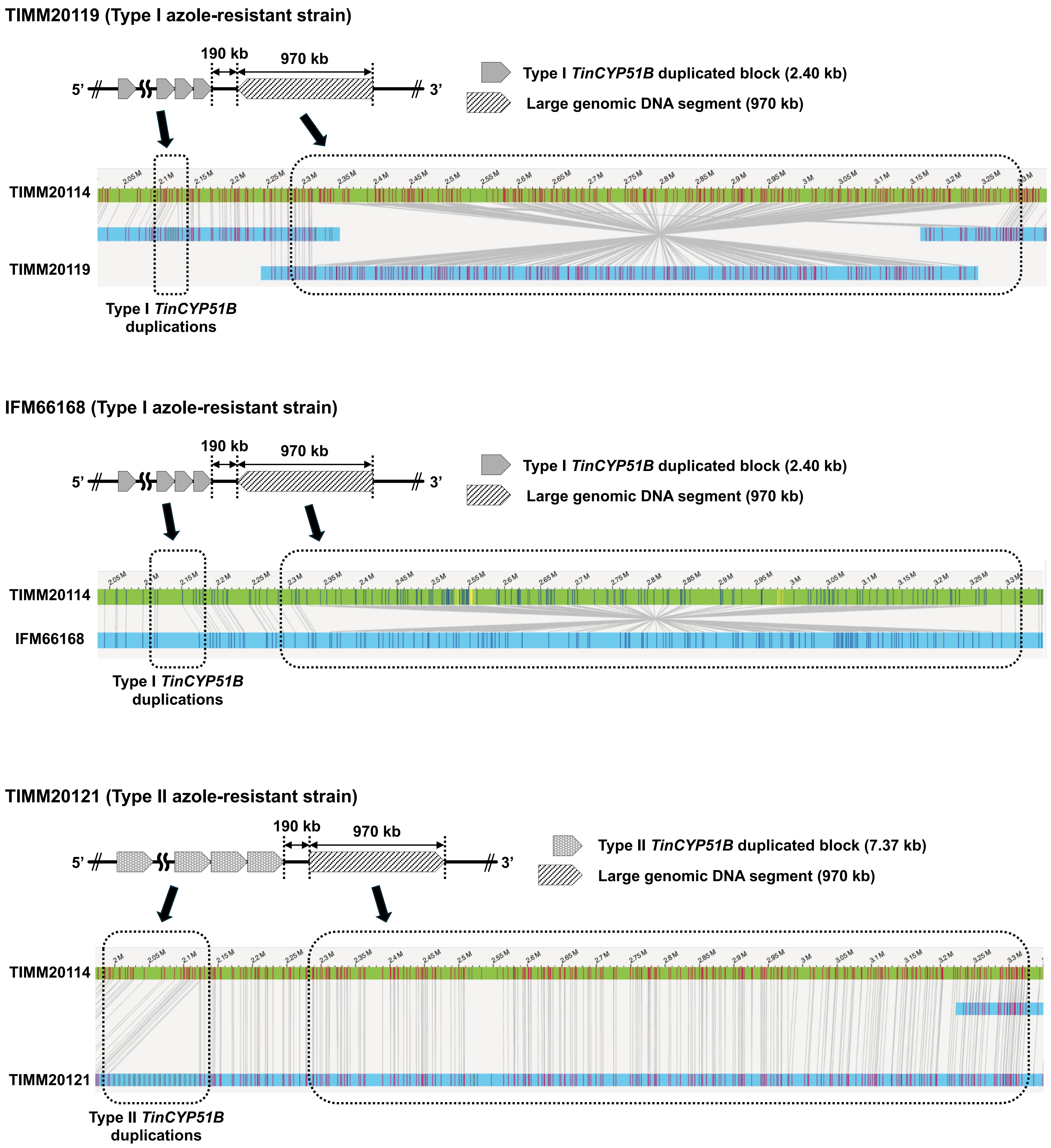

Fig. S2. The approximately 970 kb large gDNA inversion located about 190 kb downstream of the *TinCYP51B* locus in the type I azole-resistant *T. indotineae* strain TIMM20119 and IFM66168 was visualized by OGM. The large gDNA inversion was not recognized in the type II azole-resistant *T. indotineae* strain TIMM20121.
