# Supplementary material for: The entire CYP51B locus in azole-resistant isolates of the dermatophyte Trichophyton indotineae revealed by optical genome mapping
Source: Antimicrob Agents Chemother. 2026 Mar 31;70(5):e01817-25. doi: 10.1128/aac.01817-25 (PMC13148020; doi:10.1128/aac.01817-25)
Supplement: Fig. S3 — Susceptibilities to ITC and VRC of the strains 250150/18, 600098/19, 600113/19, and 600126/19 compared to TIMM 20114 (the susceptible strain) and TIMM 20119 and TIMM 2020122 (type I and type II resistant strains, respectively). [file aac.01817-25-s0003.pdf]

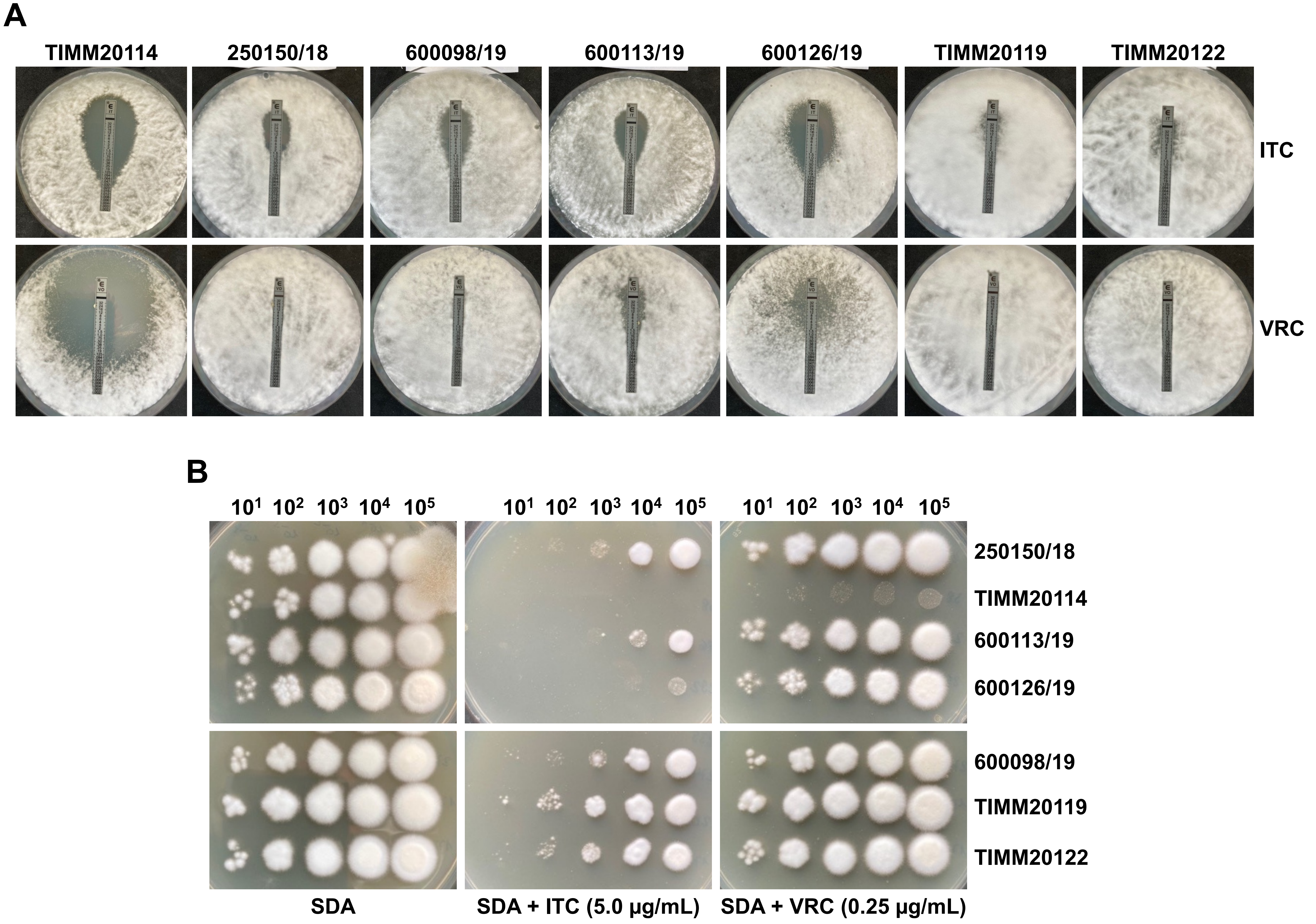

Fig. S3. Susceptibilities to ITC and VRC of the strains 250150/18, 600098/19, 600113/19, and 600126/19 compared to TIMM 20114 (the susceptible strain) and TIMM20119 and TIMM 2020122 (type I and type II resistant strains, respectively). Susceptibilities were evaluated by Etests (A) and serial dilution drug susceptibility assays (B). For the serial dilution drug susceptibility assays, spores from each strain were spotted at different dilutions on SDA plates, as previously described **(5,36)**. The plates were incubated at 28° C for six to seven days.
