# Supplementary material for: The entire CYP51B locus in azole-resistant isolates of the dermatophyte Trichophyton indotineae revealed by optical genome mapping
Source: Antimicrob Agents Chemother. 2026 Mar 31;70(5):e01817-25. doi: 10.1128/aac.01817-25 (PMC13148020; doi:10.1128/aac.01817-25)
Supplement: Fig. S4 — Southern blotting analysis of gDNA samples from T. indotineae strains. [file aac.01817-25-s0004.pdf]

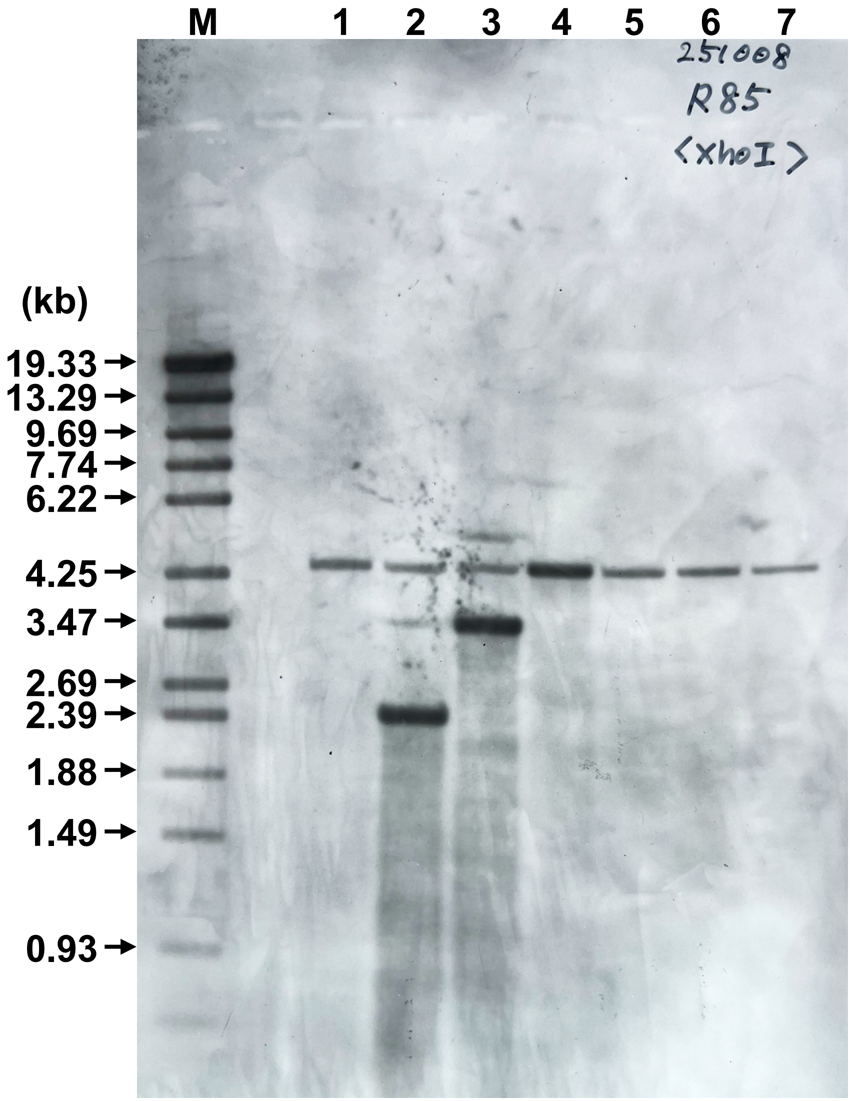

Fig. S4. Southern blotting analysis of gDNA samples from *T. indotineae* strains. Aliquots of approximately 10 μg of gDNA from each strain were digested with XhoI and separated by electrophoresis on a 0.8% (w/v) agarose gel. Lanes 1 to 7, TIMM 20114 (the susceptible strain), TIMM20119 (the type I resistant strain) and TIMM 2020121 (the type II resistant strain), 250150/18, 600098/19, 600113/19, and 600126/19, respectively. An internal fragment (approximately 410 bp) of the *TinCYP51B* gene was amplified by PCR with P16-P17 primers (Table S3) and used as a hybridization probe. The DNA standard fragment sizes are shown on the left.
