# Supplementary material for: The entire CYP51B locus in azole-resistant isolates of the dermatophyte Trichophyton indotineae revealed by optical genome mapping
Source: Antimicrob Agents Chemother. 2026 Mar 31;70(5):e01817-25. doi: 10.1128/aac.01817-25 (PMC13148020; doi:10.1128/aac.01817-25)
Supplement: Table S1 — Phenotypic and genotypic characteristics of T. indotineae strains used in this study. [file aac.01817-25-s0005.pdf]

TABLE S1 Phenotypic and genotypic characteristics of *T. indotineae* strains used in this study

Lanes (L to R) in agarose gel electrophoresis in Fig. 6C	*T. indotineae* strains[a]	Strain type	Large genomic inversion (970 kb)	ITC MIC_{80} (μg/mL)	VRC MIC_{80} (μg/mL)	Comments[b]
**1**	IFM66168 (**NUB19006**[T])	Type I	+	0.5	0.5	R
**2**	TIMM20119 (**200123/18**; IFM 67097)	Type I	+	1.0	1.0	R
**3**	**202953/19**	Type I	+	0.5	0.25	R
**4**	**205667/19**	Type II	-	0.5	0.5	R
**5**	**216510/17**	Type I	+	0.5	1.0	R
**6**	**250064/18**	Type II	-	0.25	0.5	R
**7**	TIMM20120 (**250082/18**)	Type II	-	0.25	1.0	R
**8**	TIMM20121 (**250084/18**)	Type II	-	0.5	1	R
**9**	**250101/18**	Type II	-	NA[b]	NA	
**10**	TIMM20122 (**250108/18**)	Type II	-	0.5	0.5	R
**11**	**250118/18** (**UKJ 1374/18**)	Type II	-	0.25	0.5	R
**12**	**250136/18** (**UKJ 1392/18**)	Type II	-	0.25	0.5	R
**13**	**250145/18** (**UKJ 1401/18**)	Type II	-	0.25	0.5	R
**14**	**250150/18** (**UKJ 1406/18**)	1 *TinCYP51B* gene	-	0.25	0.5	R
**15**	**250158/18** (**UKJ 1414/18**)	Type II	-	0.25	0.5	R
**16**	TIMM20114 (**UKJ 1676/17**; IFM 67092)	1 *TinCYP51B* gene	-	0.06	0.015	S
**17**	TIMM20118 (**UKJ 1687/17**; IFM 67096)	Type I	+	0.5	1.0	R
**18**	**UKJ 1691/17**	1 *TinCYP51B* gene	+	0.06	0.063	S
**19**	TIMM20115 (**UKJ 1700/17**; IFM 67093)	1 *TinCYP51B* gene	+	0.06	0.03	S
**20**	**600028/19**	Type II	-	0.25	0.5	R
**21**	**600031/19**	Type II	-	0.25	0.5	R
**22**	**600035/19**	Type II	-	0.25	0.5	R
**23**	**600047/19**	Type II	-	0.5	0.25	R
**24**	**600061/19**	Type II	-	0.25	0.5	R
**25**	**600064/19**	Type II	-	0.25	0.5	R
**26**	**600070/19**	Type II	-	0.25	0.5	R
**27**	**600071/19**	Type II	-	0.5	0.5	R
**28**	**600077/19**	Type II	-	0.5	0.5	R
**29**	**600084/19**	Type II	-	0.5	0.5	R
**30**	**600093/19**	Type II	-	0.5	1.0	R
**31**	**600094/19**	Type II	-	0.25	0.5	R
**32**	TIMM20123 (**600097/19**)	Type II	-	1.0	0.5	R
**33**	**600098/19**	1 *TinCYP51B* gene	+	1.0	0.25	R
**34**	**600103/19**	Type II	-	0.5	0.25	R
**35**	**600108/19**	Type II	-	0.5	0.5	R
**36**	**600113/19**	1 *TinCYP51B* gene	-	0.5	0.125	R
**37**	**600125/19**	Type II	-	1.0	0.5	R
**38**	**600126/19**	1 *TinCYP51B* gene	-	0.5	0.125	R
**39**	**600128/19**	Type II	-	1.0	0.5	R
**40**	**600140/19**	Type II	-	0.5	0.5	R

[a] IFM66168 is the type strain of *Trichophyton indotineae* **(16)**. This strain originates from Japan. All other strains were from previously published resistance studies in India, with the numbering in bold **(1)**. All the strains were then preserved in the culture collection of Teikyo University Institute of Medical Mycology (TIMM) and/or Medical Mycology Research Center, Chiba University (IFM), through the National Bio-Resource Project, Japan (http:// www.nbrp.jp/). ITC, itraconazole; VRC, voriconazole. [b]The letter R indicates azole resistance, while the letter S indicates azole susceptibility. NA, not analyzed.
