# Supplementary material for: The entire CYP51B locus in azole-resistant isolates of the dermatophyte Trichophyton indotineae revealed by optical genome mapping
Source: Antimicrob Agents Chemother. 2026 Mar 31;70(5):e01817-25. doi: 10.1128/aac.01817-25 (PMC13148020; doi:10.1128/aac.01817-25)
Supplement: Table S2 — Molecule statistics. [file aac.01817-25-s0006.pdf]

Table S2 Molecule stats

Category	Molecule stats	IFM66168	TIMM20118	TIMM20119	TIMM20121	TIMM20122
Input molecule stats (filtered*)	Total number of molecules	259,407	247,130	247,722	242,671	187,213
*Length of filtered DNA molecules are 150 kbp or longer.	Total length (Mbp)	56,198.21	50,703.07	51,070.87	48,466.75	39,670.64
	Average length (kbp)	216.64	205.17	206.16	199.72	211.9
	N50 molecule length (kbp)	204.54	196.39	198.21	192.01	202.65
	Enzyme used for a double-stranded DNA fluorescent labeling	DLE-1	Nt.BspQI	Nt.BspQI	Nt.BspQI	Nt.BspQI
	Label density (/100 kb)	9.7	16.81	16.02	16.42	16.7
	Coverage of the reference (X)	2,517.03	2,270.91	2,287.39	2,170.75	1,776.79
Molecules aligned to the reference	Total number of molecules aligned	184,598	207,925	196,148	199,375	84,549
	Fraction of molecules aligned	0.71	0.84	0.79	0.82	0.45
	Effective coverage of the reference (X)	1,319.02	1,584.02	1,433.02	1,463.04	577.31
	Average confidence	19.8	23.4	22.9	22.6	18.0
De novo assembly	Genome map count	10	26.0	30.0	27.0	44.0
	Total genome map length（Mbp）	24.79	22.74	22.62	23.11	24.15
	Genome map N50（kbp）	5.93	1.32	1.02	1.18	0.73
	Total reference length (Mbp)	22.33	22.33	21.2	22.33	22.33
	Total number of genome maps aligned (Fraction)	8 (0.8)	26 (1.0)	30 (1.0)	27 (1.0)	44 (1.0)
	Total unique aligned length (Mbp)	22.3	21.54	21.2	21.61	21.19
	Total unique aligned length/reference (%)	1	0.97	0.95	0.97	0.95
Molecules aligned to the assembly	Total number of molecules aligned	187,353	208,769	194,299	200,814	90,434
	Effective coverage of assembly (X)	1,189.41	1,476.19	1,327.24	1,357.47	544.4
	Average connfidence	21	23.9	23.2	23.1	18.8
