# Supplementary material for: The entire CYP51B locus in azole-resistant isolates of the dermatophyte Trichophyton indotineae revealed by optical genome mapping
Source: Antimicrob Agents Chemother. 2026 Mar 31;70(5):e01817-25. doi: 10.1128/aac.01817-25 (PMC13148020; doi:10.1128/aac.01817-25)
Supplement: Table S3 — Oligonucleotide primers used in this study. [file aac.01817-25-s0007.pdf]

Table S3 Oligonucleotide primers used in this study

Primer name	Sequence (5'-3')[a]	Chain	Purpose	References
P1	TTCCCTGACCCTCTTGTTTGGAACCCTCACCGATG	Sense	Multiplex PCR for typing the *TinCYP51B* duplication blocks	This study
P2	CTTGAACAGGAGCTGCTGCAGGACGTTGATAACAA	Anti-sense	Multiplex PCR for typing the *TinCYP51B* duplication blocks	This study
P3	GAGCTGCTCAGAGCAGTGTCTCACTCTTGTGCCAC	Sense	Multiplex PCR for typing the *TinCYP51B* duplication blocks	This study
P4	GCAACTTCAACTCCAAGTCGATGGCAGGCAAGATC	Anti-sense	Multiplex PCR for typing the *TinCYP51B* duplication blocks	This study
P5	GTCCTTATGCTCACGGCAGAGTG	Sense	Multiplex PCR for typing the *TinCYP51B* duplication blocks	This study
P6	CCTTCCATGCCCGAGTTGCTGGC	Anti-sense	Multiplex PCR for typing the *TinCYP51B* duplication blocks	This study
P7	CCTCAAGCTTGTCGATTCGTGATAGAGTAAGTGTC	Sense	Multiplex PCR for identifying the orientation of the 970 kb fragment within the genome	This study
P8	TGAGCCAGTTAGCTGCGAGAATGCTTCGCTCTACT	Anti-sense	Multiplex PCR for identifying the orientation of the 970 kb fragment within the genome	This study
P9	GGATAATGCAGGGCATGATGTCAGCCGATGTCCAA	Sense	Multiplex PCR for identifying the orientation of the 970 kb fragment within the genome	This study
P10	AAGTTCGTCAAGTTCGGCCT	Sense	qRT-PCR (*TinCYP51B*)	**6**
P11	GAGAGGCGGTGTAGATGGTG	Anti-sense	qRT-PCR (*TinCYP51B*)	**6**
P12	TGTTGGTGA TGAGGCACAGT	Sense	qRT-PCR (*TinACTIN*)	**6**
P13	CCATGTCATCCCAGTTTGTG	Anti-sense	qRT-PCR (*TinACTIN*)	**6**
P14	GCGTGACCCAGCCAACA	Sense	qRT-PCR (*TinGAPDH*)	**6**
P15	TTGGCACCTCCCTTCAAGT	Anti-sense	qRT-PCR (*TinGAPDH*)	**6**
P16	tTT*ctcgag* CCTCACCGATGGGAGAACAACGTCGG	Sense	Amplification of the *TinCYP51B*-specific hybridization probe	**6**
P17	aTta*gggccc* TCATTTCTGGGAAGGCTGCTTTCGCTTCTCCC AactcACCATTGGGTCGCCAAG	Anti-sense	Amplification of the *TinCYP51B*-specific hybridization probe	**6**

[a] Restriction sites are shown in lower case letters, italicized and underlined, and other nucleotide substituitions are shown in lower case letters.
