# Supplementary material for: The entire CYP51B locus in azole-resistant isolates of the dermatophyte Trichophyton indotineae revealed by optical genome mapping
Source: Antimicrob Agents Chemother. 2026 Mar 31;70(5):e01817-25. doi: 10.1128/aac.01817-25 (PMC13148020; doi:10.1128/aac.01817-25)
Supplement: Table S4 — Summary of results on sequencing of the full-length transcripts in the two T. indotineae strains using PacBio Iso-seq RNA long-read sequencing method. [file aac.01817-25-s0008.pdf]

Table S4 Summary of results on sequencing of the full-length transcripts in the two *T. indotineae* strains using PacBio Iso-seq RNA long-read sequencing method.

Strain no.	Strain type	Total number of reads	Average sequence length of reads (bp)	Total sequence length(bp)	>= Q30(%)[a]
TIMM20119	Type I	5,904,723	2,311	13,645,105,776	98.12
TIMM20122	Type II	4,961,269	2,228	11,052,550,497	98.16

[a] Values indicate percentage of data with a basecall accuracy of 99.9% or higher.
