# Supplementary material for: The entire CYP51B locus in azole-resistant isolates of the dermatophyte Trichophyton indotineae revealed by optical genome mapping
Source: Antimicrob Agents Chemother. 2026 Mar 31;70(5):e01817-25. doi: 10.1128/aac.01817-25 (PMC13148020; doi:10.1128/aac.01817-25)
Supplement: Table S5 — Expression of TinCYP51B in T. indotineae strains with reduced azole susceptibility. [file aac.01817-25-s0009.pdf]

TABLE S5 Expression of *TinCYP51B* in *T. indotineae* strains with reduced azole susceptibility

Lanes (L to R) in agarose gel electrophoresis in Fig. 6C	*T. indotineae* strains	ITC MIC_{80} (µg/mL)	VRC MIC_{80} (µg/mL)	Fold expression of *TinCYP51B* (mean ± SD)[a]
16	TIMM20114	0.06	0.015	1
14	250150/18	0.25	0.5	8.6 ± 1.2
33	600098/19	1	0.25	7.1 ± 1.1
36	600113/19	0.5	0.125	2.7 ± 0.2
38	600126/19	0.5	0.125	1.0 ± 0.0

[a]Results represent expression levels from three independent real-time PCR experiments. Expression levels of *TinCYP51B* genes were indicated as relative fold changes compared to the ΔCt mean of the data from TIMM20114 (control with a single copy of *TinCYP51B*). SD, standard deviation. ITC, itraconazole; VRC, voriconazole.
