# Supplementary material for: The entire CYP51B locus in azole-resistant isolates of the dermatophyte Trichophyton indotineae revealed by optical genome mapping
Source: Antimicrob Agents Chemother. 2026 Mar 31;70(5):e01817-25. doi: 10.1128/aac.01817-25 (PMC13148020; doi:10.1128/aac.01817-25)
Supplement: File S1 — The cDNA reads in the type I azole-resistant strain TIMM20119 and the type II-resistant strain TIMM20122. [file aac.01817-25-s0010.pdf]

Read no. m84079_250516_024014_s4/142738117/ccs, including the two *TinCYP51B* CDSs and polyadenylation [poly (A)] signal in TIMM20119 (read_6).

This cDNA read starts with the short 5'-upstream sequence and *TinCYP51B* CDS lacking the last five codons, followed by the *TinCYP51B* CDS lacking the last five codons with the long 5'-upstream sequence, and ends with a part of the long 5'-upstream sequence. Some region of the long 5'-upstream sequence located in the center of the read including two *TinCYP51B* CDSs is recognized as an intron (70 bp).

>TIMM20119_Read 6-intron_m84079_250516_024014_s4/142738117/ccs

GGTCCGCAGCAGCTTCAAGGCATTCATCACAAACAACGAACAAGTCTTGAGCGGAAGCAGGAGGATAAGACGATCAC
CATGGGCCTCTTAGCCGACATTGTCTCTCGTTTCTGCGAGAACTGCTCGACCCTGTCCACCGCCGCGCTCGTCGCAA
GTGCCGTATCGGCTTTTATCGTCCTCTCCATTGTTATCAACGTCCTGCAGCAGCTCCTGTTCAAGGACCCTACAAAG
CCTCCGGTGGTCTTCCACTGGGTTCCGGTCATTGGAAGCACGATCTCCTATGGAATTGACCCGTACAAGTTCTTTGA
CGACTGCAAGGAGAAGTATGGAGACATCTTCACATTCATACTGCTGGGCAAGAAGACGACTGTTTTTCTCGGTACAA
AGGGAAATGATTTCATTTTGAACGGCAAGCTCAAGGATGTTTGCGCGGAGGATGTCTACTCCCCCCTCACCACCCCA
GTGTTCGGACGACATGTGGTGTATGATTGCCCAAACTCCAAGCTCATGGAGCAGAAGAAGTTCGTCAAGTTCGGCCT
CACCTCTGAAGCTCTCCGATCCTATGTCACCCTGATCACCAAGGAAGTTGAGCAGTTCTTCGAGTCCTCCCCCGTCT
TCAAGGGCGACTCCGGAGTTTTCAACGTCAGCAAGGTCATGGCTGAAATCACCATCTACACCGCCTCTCGATCTCTA
CAGGGCAAGGAGGTGCGAGGAAAGTTCGATTCCAGCTTTGCGGAACTCTACTCCGATCTCGACATGGGCTTCGCCGC
CATCAACTTCATGTTCCCATGGTTCCCCTTCCCACACAACCGCAAGCGTGACCGTGCTCAAAAGAAGATGGCCCAGG
TTTACACCGACATCATCCGTCAGCGACGTGAGGCTGGTGGAGAGAAAGACTCCGAGGACATGGTATGGAACTTGATG
TCGTCCGTGTACAAGAATGGAACGCCAATTCCAGATATCGAAGTCGCCCACATGATGATTGCTCTTCTTATGGCTGG
CCAGCACTCTTCTTCCTCCACCGGCTCCTGGATCGTTCTCCGCCTTGCCAGCCGTCCAGATATTCTCGAGGAACTCT
ACGAGGAACAGAAACGTGTTCTCGGCGAGGATCTTCCACCACTCACCTACGAATCTCTCCAGAAACTTGATCTTCAC
AACAATGTAATCAAGGAGACTCTCCGCCTCCACGCTCCCATCCACTCTATCCTCCGTGCTGTTAAATCCCCTATGCC
CGTTGAAGGAACTAACTATGTTGTCCCAACCTCTCACAACCTCCTTGCCGCTCCTGGTGTTCCCTCACGAGACCCTC
AGTACTTCCCTGACCCTCTTGTTTGGAACCCTCACCGATGGGAGAACAACGTCGGTGTCACCGTAGTCGAGGCCAGT
GAAGAAAAGACAGATTACGAATACGGTTTGGTTAGCAAGGGTGCCAACAGCCCTTACCTCCCATTCGGCTCAGGCAG
ACACAGATGCATCGGTGAACAATTTGCATATGTTCAGCTTGGAACCGTAACAGCTACGTTAGCCAGACTAATGAAAT
GGAAGCAAGTTGAAGGCACCAAAGATGTTGTGCCGCCAACTGACTATTCGTCCCTCTTCTCGAAGCCCCTTGGCGAC
CCAATGGTCTCGTGGGAGAAGCGAAAGCAGCAGAAAAACCGGCTTGGTCGCTGCCTTGGAGACGACGGCGATTCTCG
TGTTTAGGATTTCATTTTTGAGTGATATAACTATGGGTACAATAGCGTCCACTGGGCAATGTAATTACAGTCTCTTC
TCACTCACCACCGGACAATAATAACAATATTAATATTGACGGACCGTTGATAGCTTAACCTGCCATGACTAAGCCGG
CCTCCCTGAAGCCGCGATGGCCTGATGATGCGCCCACCGTCAGGTCTCGCTCGCTCAGGCAGCAGAGAATCCAGCGG
AGGAGATGACGCCCTGCACGAACACCGTCCAGGGACCGTCTCCGCCCCGTCGCGAGCTCGAATTGTAATCATCTCGA
CGGCCGTTGGCCTGCTCGCCGCAAGGGAGAAAAAGGGGGGCAGAAGGGCAGCGGTAATTTTGGTATTGGAGATGCAG
CTGTAAACCAATAAAAAACGACGTAAAATACCAGGAACAATGCATTATCTTACAGTACCGTACGCCCCTTGCATAAA
ATGCGAGTTCGATCTCAAGATGCAACTTTTTTTTTCCCTTGCTTCGTCCGCAGCAGCTTCAAGGCATTCATCACAAA
CAACGAACAAGTCTTGAGCGGAAGCAGGAGGATAAGACGATCACCATGGGCCTCTTAGCCGACATTGTCTCTCGTTT
CTGCGAGAACTGCTCGACCCTGTCCACCGCCGCGCTCGTCGCAAGTGCCGTATCGGCTTTTATCGTCCTCTCCATTG
TTATCAACGTCCTGCAGCAGCTCCTGTTCAAGGACCCTACAAAGCCTCCGGTGGTCTTCCACTGGGTTCCGGTCATT
GGAAGCACGATCTCCTATGGAATTGACCCGTACAAGTTCTTTGACGACTGCAAGGAGAAGTATGGAGACATCTTCAC
ATTCATACTGCTGGGCAAGAAGACGACTGTTTTTCTCGGTACAAAGGGAAATGATTTCATTTTGAACGGCAAGCTCA
AGGATGTTTGCGCGGAGGATGTCTACTCCCCCCTCACCACCCCAGTGTTCGGACGACATGTGGTGTATGATTGCCCA
AACTCCAAGCTCATGGAGCAGAAGAAGTTCGTCAAGTTCGGCCTCACCTCTGAAGCTCTCCGATCCTATGTCACCCT
GATCACCAAGGAAGTTGAGCAGTTCTTCGAGTCCTCCCCCGTCTTCAAGGGCGACTCCGGAGTTTTCAACGTCAGCA
AGGTCATGGCTGAAATCACCATCTACACCGCCTCTCGATCTCTACAGGGCAAGGAGGTGCGAGGAAAGTTCGATTCC
AGCTTTGCGGAACTCTACTCCGATCTCGACATGGGCTTCGCCGCCATCAACTTCATGTTCCCATGGTTCCCCTTCCC
ACACAACCGCAAGCGTGACCGTGCTCAAAAGAAGATGGCCCAGGTTTACACCGACATCATCCGTCAGCGACGTGAGG
CTGGTGGAGAGAAAGACTCCGAGGACATGGTATGGAACTTGATGTCGTCCGTGTACAAGAATGGAACGCCAATTCCA
GATATCGAAGTCGCCCACATGATGATTGCTCTTCTTATGGCTGGCCAGCACTCTTCTTCCTCCACCGGCTCCTGGAT
CGTTCTCCGCCTTGCCAGCCGTCCAGATATTCTCGAGGAACTCTACGAGGAACAGAAACGTGTTCTCGGCGAGGATC
TTCCACCACTCACCTACGAATCTCTCCAGAAACTTGATCTTCACAACAATGTAATCAAGGAGACTCTCCGCCTCCAC
GCTCCCATCCACTCTATCCTCCGTGCTGTTAAATCCCCTATGCCCGTTGAAGGAACTAACTATGTTGTCCCAACCTC
TCACAACCTCCTTGCCGCTCCTGGTGTTCCCTCACGAGACCCTCAGTACTTCCCTGACCCTCTTGTTTGGAACCCTC
ACCGATGGGAGAACAACGTCGGTGTCACCGTAGTCGAGGCCAGTGAAGAAAAGACAGATTACGAATACGGTTTGGTT
AGCAAGGGTGCCAACAGCCCTTACCTCCCATTCGGCTCAGGCAGACACAGATGCATCGGTGAACAATTTGCATATGT

TCAGCTTGGAACCGTAACAGCTACGTTAGCCAGACTAATGAAATGGAAGCAAGTTGAAGGCACCAAAGATGTTGTGC
CGCCAACTGACTATTCGTCCCTCTTCTCGAAGCCCCTTGGCGACCCAATGGTCTCGTGGGAGAAGCGAAAGCAGCAG
AAAAACCGGCTTGGTCGCTGCCTTGGAGACGACGGCGATTCTCGTGTTTAGGATTTCATTTTTGAGTGATATAACTA
TGGGTACAATAGCGTCCACTGGGCAATGTAATTACAGTCTCTTCTCACTCACCACCGGACAATAATAACAATATTAA
TATTGACGGACCAAAAAAAAAAAAAAAAAAAAAAAAAAAAAA

Grey: Sequence upstream of *TinCYP51B* ORF
Sky blue: *TinCYP51B* complete ORF (Stop codon less)s
Yellow: Intron

Read no. m84079_250516_024014_s4/28313445/ccs, including two *TinCYP51B* CDS and the polyadenylation signal [poly(A)] in TIMM20119 (read_1).

This cDNA read starts with the short 5'-upstream sequence and *TinCYP51B* CDS lacking the last five codons, followed by the *TinCYP51B* CDS lacking the last five codons with the long 5'-upstream sequence, and ends with a part of the long 5'-upstream sequence.

>TIMM20119_Read 1_m84079_250516_024014_s4/28313445/ccs
GAAAACAACGAACAAGTCTTGAGCGGAAGCAGGAGGATAAGACGATCACCATGGGCCTCTTAGCCGACATTGTCTCT
CGTTTCTGCGAGAACTGCTCGACCCTGTCCACCGCCGCGCTCGTCGCAAGTGCCGTATCGGCTTTTATCGTCCTCTC
CATTGTTATCAACGTCCTGCAGCAGCTCCTGTTCAAGGACCCTACAAAGCCTCCGGTGGTCTTCCACTGGGTTCCGG
TCATTGGAAGCACGATCTCCTATGGAATTGACCCGTACAAGTTCTTTGACGACTGCAAGGAGAAGTATGGAGACATC
TTCACATTCATACTGCTGGGCAAGAAGACGACTGTTTTTCTCGGTACAAAGGGAAATGATTTCATTTTGAACGGCAA
GCTCAAGGATGTTTGCGCGGAGGATGTCTACTCCCCCCTCACCACCCCAGTGTTCGGACGACATGTGGTGTATGATT
GCCCAAACTCCAAGCTCATGGAGCAGAAGAAGTTCGTCAAGTTCGGCCTCACCTCTGAAGCTCTCCGATCCTATGTC
ACCCTGATCACCAAGGAAGTTGAGCAGTTCTTCGAGTCCTCCCCCGTCTTCAAGGGCGACTCCGGAGTTTTCAACGT
CAGCAAGGTCATGGCTGAAATCACCATCTACACCGCCTCTCGATCTCTACAGGGCAAGGAGGTGCGAGGAAAGTTCG
ATTCCAGCTTTGCGGAACTCTACTCCGATCTCGACATGGGCTTCGCCGCCATCAACTTCATGTTCCCATGGTTCCCC
TTCCCACACAACCGCAAGCGTGACCGTGCTCAAAAGAAGATGGCCCAGGTTTACACCGACATCATCCGTCAGCGACG
TGAGGCTGGTGGAGAGAAAGACTCCGAGGACATGGTATGGAACTTGATGTCGTCCGTGTACAAGAATGGAACGCCAA
TTCCAGATATCGAAGTCGCCCACATGATGATTGCTCTTCTTATGGCTGGCCAGCACTCTTCTTCCTCCACCGGCTCC
TGGATCGTTCTCCGCCTTGCCAGCCGTCCAGATATTCTCGAGGAACTCTACGAGGAACAGAAACGTGTTCTCGGCGA
GGATCTTCCACCACTCACCTACGAATCTCTCCAGAAACTTGATCTTCACAACAATGTAATCAAGGAGACTCTCCGCC
TCCACGCTCCCATCCACTCTATCCTCCGTGCTGTTAAATCCCCTATGCCCGTTGAAGGAACTAACTATGTTGTCCCA
ACCTCTCACAACCTCCTTGCCGCTCCTGGTGTTCCCTCACGAGACCCTCAGTACTTCCCTGACCCTCTTGTTTGGAA
CCCTCACCGATGGGAGAACAACGTCGGTGTCACCGTAGTCGAGGCCAGTGAAGAAAAGACAGATTACGAATACGGTT
TGGTTAGCAAGGGTGCCAACAGCCCTTACCTCCCATTCGGCTCAGGCAGACACAGATGCATCGGTGAACAATTTGCA
TATGTTCAGCTTGGAACCGTAACAGCTACGTTAGCCAGACTAATGAAATGGAAGCAAGTTGAAGGCACCAAAGATGT
TGTGCCGCCAACTGACTATTCGTCCCTCTTCTCGAAGCCCCTTGGCGACCCAATGGTCTCGTGGGAGAAGCGAAAGC
AGCAGAAAAACCGGCTTGGTCGCTGCCTTGGAGACGACGGCGATTCTCGTGTTTAGGATTTCATTTTGAGTGATATA
ACTATGGGTACAATAGCGTCCACTGGGCAATCTTAACCTGCCATGACTAAGCCGGCCTCCCTGAAGCCGCGATGGCC
TGATGATGCGCCCACCGTCAGGTCTCGCTCGCTCAGGCAGCAGAGAATCCAGCGGAGGAGATGACGCCCTGCACGAA
CACCGTCCAGGGACCGTCTCCGCCCCGTCGCGAGCTCGAATTGTAATCATCTCGACGGCCGTTGGCCTGCTCGCCGC
AAGGGAGAAAAAGGGGGGCAGAAGGGCAGCGGTAATTTTGGTATTGGAGATGCAGCTGTAAACCAATAAAAAACGAC
GTAAAATACCAGGAACAATGCATTATCTTACAGTACCGTACGCCCCTTGCATAAAATGCGAGTTCGATCTCAAGATG
CAACTTTTTTTTTCCCTTGCTTCGTCCGCAGCAGCTTCAAGGCATTCATCACAAACAACGAACAAGTCTTGAGCGGA
AGCAGGAGGATAAGACGATCACCATGGGCCTCTTAGCCGACATTGTCTCTCGTTTCTGCGAGAACTGCTCGACCCTG
TCCACCGCCGCGCTCGTCGCAAGTGCCGTATCGGCTTTTATCGTCCTCTCCATTGTTATCAACGTCCTGCAGCAGCT
CCTGTTCAAGGACCCTACAAAGCCTCCGGTGGTCTTCCACTGGGTTCCGGTCATTGGAAGCACGATCTCCTATGGAA
TTGACCCGTACAAGTTCTTTGACGACTGCAAGGAGAAGTATGGAGACATCTTCACATTCATACTGCTGGGCAAGAAG
ACGACTGTTTTTCTCGGTACAAAGGGAAATGATTTCATTTTGAACGGCAAGCTCAAGGATGTTTGCGCGGAGGATGT
CTACTCCCCCCTCACCACCCCAGTGTTCGGACGACATGTGGTGTATGATTGCCCAAACTCCAAGCTCATGGAGCAGA
AGAAGTTCGTCAAGTTCGGCCTCACCTCTGAAGCTCTCCGATCCTATGTCACCCTGATCACCAAGGAAGTTGAGCAG
TTCTTCGAGTCCTCCCCCGTCTTCAAGGGCGACTCCGGAGTTTTCAACGTCCAGCAAGGTCATGGCTGAAATCACCA
TCTACACCGCCTCTCGATCTCTACAGGGCAAGGAGGTGCGAGGAAAGTTCGATTCCAGCTTTGCGGAACTCTACTCC
GATCTCGACATGGGCTTCGCCGCCATCAACTTCATGTTCCCATGGTTCCCCTTCCCACACAACCGCAAGCGTGACCG
TGCTCAAAAGAAGATGGCCCAGGTTTACACCGACATCATCCGTCAGCGACGTGAGGCTGGTGGAGAGAAAGACTCCG

AGGACATGGTATGGAACTTGATGTCGTCCGTGTACAAGAATGGAACGCCAATTCCAGATATCGAAGTCGCCCACATG
ATGATTGCTCTTCTTATGGCTGGCCAGCACTCTTCTTCCTCCACCGGCTCCTGGATCGTTCTCCGCCTTGCCAGCCG
TCCAGATATTCTCGAGGAACTCTACGAGGAACAGAAACGTGTTCTCGGCGAGGATCTTCCACCACTCACCTACGAAT
CTCTCCAGAAACTTGATCTTCACAACAATGTAATCAAGGAGACTCTCCGCCTCCACGCTCCCATCCACTCTATCCTC
CGTGCTGTTAAATCCCCTATGCCCGTTGAAGGAACTAACTATGTTGTCCCAACCTCTCACAACCTCCTTGCCGCTCC
TGGTGTTCCCTCACGAGACCCTCAGTACTTCCCTGACCCTCTTGTTTGGAACCCTCACCGATGGGAGAACAACGTCG
GTGTCACCGTAGTCGAGGCCAGTGAAGAAAAGACAGATTACGAATACGGTTTGGTTAGCAAGGGTGCCAACAGCCCT
TACCTCCCATTCGGCTCAGGCAGACACAGATGCATCGGTGAACAATTTGCATATGTTCAGCTTGGAACCGTAACAGC
TACGTTAGCCAGACTAATGAAATGGAAGCAAGTTGAAGGCACCAAAGATGTTGTGCCGCCAACTGACTATTCGTCCT
CTTCTCGAAGCCCCTTGGCGACCCAATGGTCTCGTGGGAGAAGCGAAAGCAGCAGAAAAACCGGCTTGGTCGCTGCC
TTGGAGACGACGGCGATTCTCGTGTTTAGGATTTCATTTTTGAGTGATATAACTATGGGTACAATAGCGTCCACTGG
GCAATCTTAACCTGCCATGACTAAGCCGGCCTCCCTGAAGCCCGCGATGGCCTGATGATGCGCCCACCGTCAGGTCT
CGCTCGCTCAGGCAGCAGAGAATCCAGCGGAGGAGATGACGCCCTGCACGAACACCGTCCAGGGACCGTCTCCGCCC
CGTCGCGAGCTCGAATTGTAATCATCTCGACGGCCGTTGGCCTGCTCGCCGCAAGGGAGAAAAAGGGGGGCAGAAGG
GCAGCGGTAATTTTGGTATTGGAGATGCAGCTGTAAACCAATAAAAAACGACGTAAAATACCAGGAACAATGCATTA
TCTTACAGTACCGTAAAAAAAAAAAAAAAAAAA

Grey: Sequence upstream of *TinCYP51B* ORF
Sky blue: *TinCYP51B* complete ORF (Stop codon less)

Read no. m84079_250516_024014_s4/135463143/ccs, including the two *TinCYP51B* CDSs and polyadenylation [poly (A)] signal in TIMM20119 (read_2).

This cDNA read starts with the short 5'-upstream sequence and *TinCYP51B* CDS lacking the last five codons, followed by the intact *TinCYP51B* CDS with the long 5'-upstream sequence, and ends with the 3'-downstream sequence.

>TIMM20119_Read 2_m84079_250516_024014_s4/135463143/ccs
GGTCCGCAGCAGCTTCAAGGCATTCATCACAAACAACGAACAAGTCTTGAGCGGAAGCAGGAGGATAAGACGATCAC
CATGGGCCTCTTAGCCGACATTGTCTCTCGTTTCTGCGAGAACTGCTCGACCCTGTCCACCGCCGCGCTCGTCGCAA
GTGCCGTATCGGCTTTTATCGTCCTCTCCATTGTTATCAACGTCCTGCAGCAGCTCCTGTTCAAGGACCCTACAAAG
CCTCCGGTGGTCTTCCACTGGGTTCCGGTCATTGGAAGCACGATCTCCTATGGAATTGACCCGTACAAGTTCTTTGA
CGACTGCAAGGAGAAGTATGGAGACATCTTCACATTCATACTGCTGGGCAAGAAGACGACTGTTTTTCTCGGTACAA
AGGGAAATGATTTCATTTTGAACGGCAAGCTCAAGGATGTTTGCGCGGAGGATGTCTACTCCCCCCTCACCACCCCA
GTGTTCGGACGACATGTGGTGTATGATTGCCCAAACTCCAAGCTCATGGAGCAGAAGAAGTTCGTCAAGTTCGGCCT
CACCTCTGAAGCTCTCCGATCCTATGTCACCCTGATCACCAAGGAAGTTGAGCAGTTCTTCGAGTCCTCCCCCGTCT
TCAAGGGCGACTCCGGAGTTTTCAACGTCAGCAAGGTCATGGCTGAAATCACCATCTACACCGCCTCTCGATCTCTA
CAGGGCAAGGAGGTGCGAGGAAAGTTCGATTCCAGCTTTGCGGAACTCTACTCCGATCTCGACATGGGCTTCGCCGC
CATCAACTTCATGTTCCCATGGTTCCCCTTCCCACACAACCGCAAGCGTGACCGTGCTCAAAAGAAGATGGCCCAGG
TTTACACCGACATCATCCGTCAGCGACGTGAGGCTGGTGGAGAGAAAGACTCCGAGGACATGGTATGGAACTTGATG
TCGTCCGTGTACAAGAATGGAACGCCAATTCCAGATATCGAAGTCGCCCACATGATGATTGCTCTTCTTATGGCTGG
CCAGCACTCTTCTTCCTCCACCGGCTCCTGGATCGTTCTCCGCCTTGCCAGCCGTCCAGATATTCTCGAGGAACTCT
ACGAGGAACAGAAACGTGTTCTCGGCGAGGATCTTCCACCACTCACCTACGAATCTCTCCAGAAACTTGATCTTCAC
AACAATGTAATCAAGGAGACTCTCCGCCTCCACGCTCCCATCCACTCTATCCTCCGTGCTGTTAAATCCCCTATGCC
CGTTGAAGGAACTAACTATGTTGTCCCAACCTCTCACAACCTCCTTGCCGCTCCTGGTGTTCCCTCACGAGACCCTC
AGTACTTCCCTGACCCTCTTGTTTGGAACCCTCACCGATGGGAGAACAACGTCGGTGTCACCGTAGTCGAGGCCAGT
GAAGAAAAGACAGATTACGAATACGGTTTGGTTAGCAAGGGTGCCAACAGCCCTTACCTCCCATTCGGCTCAGGCAG
ACACAGATGCATCGGTGAACAATTTGCATATGTTCAGCTTGGAACCGTAACAGCTACGTTAGCCAGACTAATGAAAT
GGAAGCAAGTTGAAGGCACCAAAGATGTTGTGCCGCCAACTACTATTCGTCCCTCTTCTCGAAGCCCCTTGGCGACC
CAATGGTCTCGTGGGAGAAGCGAAAGCAGCAGAAAAACCGGCTTGGTCGCTGCCTTGGAGACGACGGCGATTCTCGT
GTTTAGGATTTCATTTTTGAGTGATATAACTATGGGTACAATAGCGTCCACTGGGCAATCTTAACCTGCCATGACTA
AGCCGGCCTCCCTGAAGCCGCGATGGCCTGATGATGCGCCCACCGTCAGGTCTCGCTCGCTCAGGCAGCAGAGAATC
CAGCGGAGGAGATGACGCCCTGCACGAACACCGTCCAGGGACCGTCTCCGCCCCGTCGCGAGCTCGAATTGTAATCA
TCTCGACGGCCGTTGGCCTGCTCGCCGCAAGGGAGAAAAAGGGGGGCAGAAGGGCAGCGGTAATTTTGGTATTGGAG
ATGCAGCTGTAAACCAATAAAAAACGACGTAAAATACCAGGAACAATGCATTATCTTACAGTACCGTACGCCCCTTG
CATAAAATGCGAGTTCGATCTCAAGATGCAACTTTTTTTTTCCCTTGCTTCGTCCGCAGCAGCTTCAAGGCATTCAT
CACAAACAACGAACAAGTCTTGAGCGGAAGCAGGAGGATAAGACGATCACCATGGGCCTCTTAGCCGACATTGTCTC

TCGTTTCTGCGAGAACTGCTCGACCCTGTCCACCGCCGCGCTCGTCGCAAGTGCCGTATCGGCTTTTATCGTCCTCT
CCATTGTTATCAACGTCCTGCAGCAGCTCCTGTTCAAGGACCCTACAAAGCCTCCGGTGGTCTTCCACTGGGTTCCG
GTCATTGGAAGCACGATCTCCTATGGAATTGACCCGTACAAGTTCTTTGACGACTGCAAGGAGAAGTATGGAGACAT
CTTCACATTCATACTGCTGGGCAAGAAGACGACTGTTTTTCTCGGTACAAAGGGAAATGATTTCATTTTGAACGGCA
AGCTCAAGGATGTTTGCGCGGAGGATGTCTACTCCCCCCTCACCACCCCAGTGTTCGGACGACATGTGGTGTATGAT
TGCCCAAACTCCAAGCTCATGGAGCAGAAGAAGTTCGTCAAGTTCGGCCTCACCTCTGAAGCTCTCCGATCCTATGT
CACCCTGATCACCAAGGAAGTTGAGCAGTTCTTCGAGTCCTCCCCCGTCTTCAAGGGCGACTCCGGAGTTTTCAACG
TCAGCAAGGTCATGGCTGAAATCACCATCTACACCGCCTCTCGATCTCTACAGGGCAAGGAGGTGCGAGGAAAGTTC
GATTCCAGCTTTGCGGAACTCTACTCCGATCTCGACATGGGCTTCGCCGCCATCAACTTCATGTTCCCATGGTTCCC
CTTCCCACACAACCGCAAGCGTGACCGTGCTCAAAAGAAGATGGCCCAGGTTTACACCGACATCATCCGTCAGCGAC
GTGAGGCTGGTGGAGAGAAAGACTCCGAGGACATGGTATGGAACTTGATGTCGTCCGTGTACAAGAATGGAACGCCA
ATTCCAGATATCGAAGTCGCCCACATGATGATTGCTCTTCTTATGGCTGGCCAGCACTCTTCTTCCTCCACCGGCTC
CTGGATCGTTCTCCGCCTTGCCAGCCGTCCAGATATTCTCGAGGAACTCTACGAGGAACAGAAACGTGTTCTCGGCG
AGGATCTTCCACCACTCACCTACGAATCTCTCCAGAAACTTGATCTTCACAACAATGTAATCAAGGAGACTCTCCGC
CTCCACGCTCCCATCCACTCTATCCTCCGTGCTGTTAAATCCCCTATGCCCGTTGAAGGAACTAACTATGTTGTCCC
AACCTCTCACAACCTCCTTGCCGCTCCTGGTGTTCCCTCACGAGACCCTCAGTACTTCCCTGACCCTCTTGTTTGGA
ACCCTCACCGATGGGAGAACAACGTCGGTGTCACCGTAGTCGAGGCCAGTGAAGAAAAGACAGATTACGAATACGGT
TTGGTTAGCAAGGGTGCCAACAGCCCTTACCTCCCATTCGGCTCAGGCAGACACAGATGCATCGGTGAACAATTTGC
ATATGTTCAGCTTGGAACCGTAACAGCTACGTTAGCCAGACTAATGAAATGGAAGCAAGTTGAAGGCACCAAAGATG
TTGTGCCGCCAACTGACTATTCGTCCCTCTTCTCGAAGCCCCTTGGCGACCCAATGGTCTCGTGGGAGAAGCGAAAG
CAGC**CTTCCCAGAAATGA**TAATCACGACTGCGGATTGAATCATGTTAAATTTCTTGCTTCTTGCTAATTTATTTCTT
CTCTTTTCTTACGTTTATACACCGAGCCATATTTGCACTTTATGGTCACGCAAATAGGTTTTCGGAATAGAGCATAT
GTAATAAAAACTTTCTTTTGACCATGCTTTTGAAGATGGGGGATGTAGATAATAGTATTATTCTAATACAGCCATTT
ATTACAAAAAAAAAAAAAAAAAAAAAAAAAAAAAAAA

Grey: Sequence upstream of *TinCYP51B* ORF
Sky blue: *TinCYP51B* complete ORF (Stop codon less)
Sky blue_pink: *TinCYP51B* complete ORF
Dark yellow: Sequence downstream of *TinCYP51B* complete ORF

Read no. m84079_250516_024014_s4/100009345/ccs, including the *TinCYP51B* CDS and polyadenylation [poly (A)] signal in TIMM20119 (read_3).

This cDNA read starts with the short 5'-upstream sequence and *TinCYP51B* CDS lacking the last five codons, and ends with a part of the long 5'-upstream sequence.

>TIMM20119_Read 3_m84079_250516_024014_s4/100009345/ccs
GGTCCGCAGCAGCTTCAAGGCATTCATCACAAACAACGAACAAGTCTTGAGCGGAAGCAGGAGGATAAGACGATCAC
CATGGGCCTCTTAGCCGACATTGTCTCTCGTTTCTGCGAGAACTGCTCGACCCTGTCCACCGCCGCGCTCGTCGCAA
GTGCCGTATCGGCTTTTATCGTCCTCTCCATTGTTATCAACGTCCTGCAGCAGCTCCTGTTCAAGGACCCTACAAAG
CCTCCGGTGGTCTTCCACTGGGTTCCGGTCATTGGAAGCACGATCTCCTATGGAATTGACCCGTACAAGTTCTTTGA
CGACTGCAAGGAGAAGTATGGAGACATCTTCACATTCATACTGCTGGGCAAGAAGACGACTGTTTTTCTCGGTACAA
AGGGAAATGATTTCATTTTGAACGGCAAGCTCAAGGATGTTTGCGCGGAGGATGTCTACTCCCCCCTCACCACCCCA
GTGTTCGGACGACATGTGGTGTATGATTGCCCAAACTCCAAGCTCATGGAGCAGAAGAAGTTCGTCAAGTTCGGCCT
CACCTCTGAAGCTCTCCGATCCTATGTCACCCTGATCACCAAGGAAGTTGAGCAGTTCTTCGAGTCCTCCCCCGTCT
TCAAGGGCGACTCCGGAGTTTTCAACGTCAGCAAGGTCATGGCTGAAATCACCATCTACACCGCCTCTCGATCTCTA
CAGGGCAAGGAGGTGCGAGGAAAGTTCGATTCCAGCTTTGCGGAACTCTACTCCGATCTCGACATGGGCTTCGCCGC
CATCAACTTCATGTTCCCATGGTTCCCCTTCCCACACAACCGCAAGCGTGACCGTGCTCAAAAGAAGATGGCCCAGG
TTTACACCGACATCATCCGTCAGCGACGTGAGGCTGGTGGAGAGAAAGACTCCGAGGACATGGTATGGAACTTGATG
TCGTCCGTGTACAAGAATGGAACGCCAATTCCAGATATCGAAGTCGCCCACATGATGATTGCTCTTCTTATGGCTGG
CCAGCACTCTTCTTCCTCCACCGGCTCCTGGATCGTTCTCCGCCTTGCCAGCCGTCCAGATATTCTCGAGGAACTCT
ACGAGGAACAGAAACGTGTTCTCGGCGAGGATCTTCCACCACTCACCTACGAATCTCTCCAGAAACTTGATCTTCAC
AACAATGTAATCAAGGAGACTCTCCGCCTCCACGCTCCCATCCACTCTATCCTCCGTGCTGTTAAATCCCCTATGCC
CGTTGAAGGAACTAACTATGTTGTCCCAACCTCTCACAACCTCCTTGCCGCTCCTGGTGTTCCCTCACGAGACCCTC
AGTACTTCCCTGACCCTCTTGTTTGGAACCCTCACCGATGGGAGAACAACGTCGGTGTCACCGTAGTCGAGGCCAGT
GAAGAAAAGACAGATTACGAATACGGTTTGGTTAGCAAGGGTGCCAACAGCCCTTACCTCCCATTCGGCTCAGGCAG
ACACAGATGCATCGGTGAACAATTTGCATATGTTCAGCTTGGAACCGTAACAGCTACGTTAGCCAGACTAATGAAAT

GGAAGCAAGTTGAAGGCACCAAAGATGTTGTGCCGCCAACTGACTATTCGTCCCTCTTCTCGAAGCCCCTTGGCGAC
CCAATGGTCTCGTGGGAGAAGCGAAAGCAGCAGAAAAACCGGCTTGGTCGCTGCCTTGGAGACGACGGCGATTCTCG
TGTTTAGGATTTCATTTTTGAGTGATATAACTATGGGTACAATAGCGTCCACTGGGCAATGTAATTACAGTCTCTTC
TCACTCACCACCGGACAATAATAACAATATTAATATTAAAAAAAAAAAAAAAAAAAAAAAAAAAAAAAA

Grey: Sequence upstream of *TinCYP51B* ORF
Sky blue_: *TinCYP51B* ORF (Stop codon less)

Read no. m84079_250516_024014_s4/10031052/ccs, including the *TinCYP51B* CDS and polyadenylation [poly (A)] signal in TIMM20119 (read_4).

This cDNA read includes a single intact *TinCYP51B* CDS with the short 5'-upstream sequence and the 3'-downstream sequence.

>TIMM20119_Read 4_m84079_250516_024014_s4/10031052/ccs
GGTCCGCAGCAGCTTCAAGGCATTCATCACAAACAACGAACAAGTCTTGAGCGGAAGCAGGAGGATAAGACGATCAC
CATGGGCCTCTTAGCCGACATTGTCTCTCGTTTCTGCGAGAACTGCTCGACCCTGTCCACCGCCGCGCTCGTCGCAA
GTGCCGTATCGGCTTTTATCGTCCTCTCCATTGTTATCAACGTCCTGCAGCAGCTCCTGTTCAAGGACCCTACAAAG
CCTCCGGTGGTCTTCCACTGGGTTCCGGTCATTGGAAGCACGATCTCCTATGGAATTGACCCGTACAAGTTCTTTGA
CGACTGCAAGGAGAAGTATGGAGACATCTTCACATTCATACTGCTGGGCAAGAAGACGACTGTTTTTCTCGGTACAA
AGGGAAATGATTTCATTTTGAACGGCAAGCTCAAGGATGTTTGCGCGGAGGATGTCTACTCCCCCCTCACCACCCCA
GTGTTCGGACGACATGTGGTGTATGATTGCCCAAACTCCAAGCTCATGGAGCAGAAGAAGTTCGTCAAGTTCGGCCT
CACCTCTGAAGCTCTCCGATCCTATGTCACCCTGATCACCAAGGAAGTTGAGCAGTTCTTCGAGTCCTCCCCCGTCT
TCAAGGGCGACTCCGGAGTTTTCAACGTCAGCAAGGTCATGGCTGAAATCACCATCTACACCGCCTCTCGATCTCTA
CAGGGCAAGGAGGTGCGAGGAAAGTTCGATTCCAGCTTTGCGGAACTCTACTCCGATCTCGACATGGGCTTCGCCGC
CATCAACTTCATGTTCCCATGGTTCCCCTTCCCACACAACCGCAAGCGTGACCGTGCTCAAAAGAAGATGGCCCAGG
TTTACACCGACATCATCCGTCAGCGACGTGAGGCTGGTGGAGAGAAAGACTCCGAGGACATGGTATGGAACTTGATG
TCGTCCGTGTACAAGAATGGAACGCCAATTCCAGATATCGAAGTCGCCCACATGATGATTGCTCTTCTTATGGCTGG
CCAGCACTCTTCTTCCTCCACCGGCTCCTGGATCGTTCTCCGCCTTGCCAGCCGTCCAGATATTCTCGAGGAACTCT
ACGAGGAACAGAAACGTGTTCTCGGCGAGGATCTTCCACCACTCACCTACGAATCTCTCCAGAAACTTGATCTTCAC
AACAATGTAATCAAGGAGACTCTCCGCCTCCACGCTCCCATCCACTCTATCCTCCGTGCTGTTAAATCCCCTATGCC
CGTTGAAGGAACTAACTATGTTGTCCCAACCTCTCACAACCTCCTTGCCGCTCCTGGTGTTCCCTCACGAGACCCTC
AGTACTTCCCTGACCCTCTTGTTTGGAACCCTCACCGATGGGAGAACAACGTCGGTGTCACCGTAGTCGAGGCCAGT
GAAGAAAAGACAGATTACGAATACGGTTTGGTTAGCAAGGGTGCCAACAGCCCTTACCTCCCATTCGGCTCAGGCAG
ACACAGATGCATCGGTGAACAATTTGCATATGTTCAGCTTGGAACCGTAACAGCTACGTTAGCCAGACTAATGAAAT
GGAAGCAAGTTGAAGGCACCAAAGATGTTGTGCCGCCAACTGACTATTCGTCCCTCTTCTCGAAGCCCCTTGGCGAC
CCAATGGTCTCGTGGGAGAAGCGAAAGCAGC**CTTCCCAGAAATGA**TAATCACGACTGCGGATTGAATCATGTTAAAT
TTCTTGCTTCTTGCTAATTTATTTCTTCTCTTTTCTTACGTTTATACACCGAGCCATATTTGCACTTTATGGTCACG
CAAATAGGTTTTCGGAATAGAGCATATGTAATAAAAACTTTCTTTTGACCATGCTTTTGAAGATGGGGGATGTAGAT
AATAGTATTATTCTAATACAGCCATTTATTACAATAAAAAAAAAAAAAAAAAAAAAAAAAAAAA

Grey: Sequence upstream of *TinCYP51B* ORF
Sky blue_pink: *TinCYP51B* complete ORF
Dark yellow: Sequence downstream of *TinCYP51B* complete ORF

Read no. m84079_250516_024014_s4/194777583/ccs, including the *TinCYP51B* CDS and polyadenylation [poly (A)] signal in TIMM20122 (read_5).

This cDNA read includes a single intact *TinCYP51B* CDS with the long 5'-upstream sequence and the 3'-downstream sequence.

>TIMM20122_Read 5_m84079_250516_024014_s4/194777583/ccs
GAATTACAGTCTCTTCTCACTCACCACCGGACAATAATAACAATATTAATATTGACGGACCGTTGATAGCTTAACCT
GCCATGACTAAGCCGGCCTCCCTGAAGCCGCGATGGCCTGATGATGCGCCCACCGTCAGGTCTCGCTCGCTCAGGCA
GCAGAGAATCCAGCGGAGGAGATGACGCCCTGCACGAACACCGTCCAGGGACCGTCTCCGCCCCGTCGCGAGCTCGA

ATTGTAATCATCTCGACGGCCGTTGGCCTGCTCGCCGCAAGGGAGAAAAAGGGGGGCAGAAGGGCAGCGGTAATTTT
GGTATTGGAGATGCAGCTGTAAACCAATAAAAAACGACGTAAAATACCAGGAACAATGCATTATCTTACAGTACCGT
ACGCCCCTTGCATAAAATGCGAGTTCGATCTCAAGATGCAACTTTTTTTTTCCCTTGCTTCGTCCGCAGCAGCTTCA
AGGCATTCATCACAAACAACGAACAAGTCTTGAGCGGAAGCAGGAGGATAAGACGATCACCATGGGCCTCTTAGCCG
ACATTGTCTCTCGTTTCTGCGAGAACTGCTCGACCCTGTCCACCGCCGCGCTCGTCGCAAGTGCCGTATCGGCTTTT
ATCGTCCTCTCCATTGTTATCAACGTCCTGCAGCAGCTCCTGTTCAAGGACCCTACAAAGCCTCCGGTGGTCTTCCA
CTGGGTTCCGGTCATTGGAAGCACGATCTCCTATGGAATTGACCCGTACAAGTTCTTTGACGACTGCAAGGAGAAGT
ATGGAGACATCTTCACATTCATACTGCTGGGCAAGAAGACGACTGTTTTTCTCGGTACAAAGGGAAATGATTTCATT
TTGAACGGCAAGCTCAAGGATGTTTGCGCGGAGGATGTCTACTCCCCCCTCACCACCCCAGTGTTCGGACGACATGT
GGTGTATGATTGCCCAAACTCCAAGCTCATGGAGCAGAAGAAGTTCGTCAAGTTCGGCCTCACCTCTGAAGCTCTCC
GATCCTATGTCACCCTGATCACCAAGGAAGTTGAGCAGTTCTTCGAGTCCTCCCCCGTCTTCAAGGGCGACTCCGGA
GTTTTCAACGTCAGCAAGGTCATGGCTGAAATCACCATCTACACCGCCTCTCGATCTCTACAGGGCAAGGAGGTGCG
AGGAAAGTTCGATTCCAGCTTTGCGGAACTCTACTCCGATCTCGACATGGGCTTCGCCGCCATCAACTTCATGTTCC
CATGGTTCCCCTTCCCACACAACCGCAAGCGTGACCGTGCTCAAAAGAAGATGGCCCAGGTTTACACCGACATCATC
CGTCAGCGACGTGAGGCTGGTGGAGAGAAAGACTCCGAGGACATGGTATGGAACTTGATGTCGTCCGTGTACAAGAA
TGGAACGCCAATTCCAGATATCGAAGTCGCCCACATGATGATTGCTCTTCTTATGGCTGGCCAGCACTCTTCTTCCT
CCACCGGCTCCTGGATCGTTCTCCGCCTTGCCAGCCGTCCAGATATTCTCGAGGAACTCTACGAGGAACAGAAACGT
GTTCTCGGCGAGGATCTTCCACCACTCACCTACGAATCTCTCCAGAAACTTGATCTTCACAACAATGTAATCAAGGA
GACTCTCCGCCTCCACGCTCCCATCCACTCTATCCTCCGTGCTGTTAAATCCCCTATGCCCGTTGAAGGAACTAACT
ATGTTGTCCCAACCTCTCACAACCTCCTTGCCGCTCCTGGTGTTCCCTCACGAGACCCTCAGTACTTCCCTGACCCT
CTTGTTTGGAACCCTCACCGATGGGAGAACAACGTCGGTGTCACCGTAGTCGAGGCCAGTGAAGAAAAGACAGATTA
CGGATACGGTTTGGTTAGCAAGGGTGCCAACAGCCCTTACCTCCCATTCGGCTCAGGCAGACACAGATGCATCGGTG
AACAATTTGCATATGTTCAGCTTGGAACCGTAACAGCTACGTTAGCCAGACTAATGAAATGGAAGCAAGTTGAAGGC
ACCAAAGATGTTGTGCCGCCAACTGACTATTCGTCCCTCTTCTCGAAGCCCCTTGGCGACCCAATGGTCTCGTGGGA
GAAGCGAAAGCAGC**CTTCCCAGAAATGA**TAATCACGACTGCGGATTGAATCATGTTAAATTTCTTGCTTCTTGCTAA
TTTATTTCTTCTCTTTTCTTACGTTTATACACCGAGCCATATTTGCACTTTATGGTCACGCAAATAGGTTTTCGGAA
TAGAGCATATGTAATAAAAACTTTCTTTTGACCATGCTTTTGAAGATGGGGGATGTAGATAATAGTATTATTCTAAT
ACAGCCATTTATTACAAAAAAAAAAAAAAAAAAAAAAAAAA

Grey: Sequence upstream of *TinCYP51B* ORF
Sky blue: *TinCYP51B* complete ORF (Stop codon less)
Sky blue_pink: *TinCYP51B* complete ORF
Dark yellow: Sequence downstream of *TinCYP51B* complete ORF
